# Discovery of Ongoing Selective Sweeps within *Anopheles* Mosquito Populations Using Deep Learning

**DOI:** 10.1093/molbev/msaa259

**Published:** 2020-10-06

**Authors:** Alexander T Xue, Daniel R Schrider, Andrew D Kern, Ag1000g Consortium, Alessandra della Torre, Andrew Kern, Beniamino Caputo, Bilali Kabula, Bradley White, Charles Godfray, Constant Edi, Craig Wilding, Dan Neafsey, Daniel Schrider, David Conway, David Weetman, Diego Ayala, Dominic Kwiatkowski, Igor Sharakhov, Janet Midega, Jiannong (John) Xu, João Pinto, John Essandoh, Johnson Matowo, Ken Vernick, Luc S Djogbenou, Mamadou Coulibaly, Mara Lawniczak, Martin Donnelly, Matthew Hahn, Michaël Fontaine, Michelle Riehle, Nora Besansky, Omar Cornejo, Robert McCann, Sam O’Loughlin, Vincent Robert, Alexander Xue, Alistair Miles, Chris Clarkson, CJ Battey, Cody Champion, Frederic Labbe, Giordano Bottà, Jeffrey Adrion, Joel Nelson, Nick Harding, Richard Wang, Scott T Small, Seth Redmond, Tiago Antão

**Affiliations:** 1 Simons Center for Quantitative Biology, Cold Spring Harbor Laboratory, Cold Spring Harbor, NY; 2 Department of Genetics, University of North Carolina, Chapel Hill, NC; 3 Institute of Ecology and Evolution, 5289 University of Oregon, Eugene, OR

**Keywords:** population genomics, selective sweeps, partial sweeps, machine learning, convolutional neural networks

## Abstract

Identification of partial sweeps, which include both hard and soft sweeps that have not currently reached fixation, provides crucial information about ongoing evolutionary responses. To this end, we introduce *partialS/HIC*, a deep learning method to discover selective sweeps from population genomic data. *partialS/HIC* uses a convolutional neural network for image processing, which is trained with a large suite of summary statistics derived from coalescent simulations incorporating population-specific history, to distinguish between completed versus partial sweeps, hard versus soft sweeps, and regions directly affected by selection versus those merely linked to nearby selective sweeps. We perform several simulation experiments under various demographic scenarios to demonstrate *partialS/HIC*’s performance, which exhibits excellent resolution for detecting partial sweeps. We also apply our classifier to whole genomes from eight mosquito populations sampled across sub-Saharan Africa by the *Anopheles gambiae* 1000 Genomes Consortium, elucidating both continent-wide patterns as well as sweeps unique to specific geographic regions. These populations have experienced intense insecticide exposure over the past two decades, and we observe a strong overrepresentation of sweeps at insecticide resistance loci. Our analysis thus provides a list of candidate adaptive loci that may be relevant to mosquito control efforts. More broadly, our supervised machine learning approach introduces a method to distinguish between completed and partial sweeps, as well as between hard and soft sweeps, under a variety of demographic scenarios. As whole-genome data rapidly accumulate for a greater diversity of organisms, *partialS/HIC* addresses an increasing demand for useful selection scan tools that can track in-progress evolutionary dynamics.

## Introduction

Malaria represents an enormous burden on human health, with an estimated 214 million cases and 438,000 deaths in 2015 (WHO 2015). As mosquitos of the *Anopheles gambiae* species complex are the major vector for *Plasmodium* parasites, roughly 70% of global malaria relief budgets have been focused on mosquito control, including insecticide-treated bed-nets, indoor residual spraying, and larva control through the direct modification of habitats as well as the application of larvicide. Although these vector control efforts have successfully produced major reductions of malaria transmission rates over the past 15 years (WHO 2015), there has been an alarming increase in mosquitos resistant to insecticides, specifically pyrethroids, observed across nearly all areas of the world covered by anti-malarial efforts (Hemingway et al. 2016). Pyrethroids are the only class of insecticide used in long-lasting insecticidal nets and are applied in many indoor spraying programs, thus the evolutionary innovation of resistance is a well-recognized Achilles heel of anti-malarial efforts.

The increase in insecticide-resistant mosquitoes is to be expected from an evolutionary standpoint: anti-malaria control efforts exert a strong selective pressure to which mosquito populations will respond through the differential survivorship and reproduction of those individuals that can best cope with the applied insecticides. Pyrethroid resistance was reported within African malaria vectors first in Sudan during the 1970s, then later in West Africa during the 1990s, most likely stemming from accidental exposure of mosquitos to crop applications of pyrethroids (Brown 1986; Elissa et al. 1993). Subsequent analysis showed this earliest resistance to be a result of mutations in the knockdown resistance locus *kdr*, which is known to contribute to pyrethroid resistance in other insect species (Martinez-Torres et al. 1998). Mutations conferring resistance at *kdr* as well as other loci have since spread throughout Africa, and threaten to nullify the gains in malaria control achieved over the past decade (Miles et al. 2017). Although control efforts are now looking toward nonpyrethroid insecticides (Oxborough et al. 2015; Hemingway et al. 2016) as well as gene drive technologies (Hammond et al. 2016), it is anticipated that resistance to these control modalities will eventually evolve as well (Unckless et al. 2015, 2017). Hence, an important goal in the continued fight against malaria is to identify genomic targets of resistance in *Anopheles*, especially in such a way that might inform vector managers in the field.

Alleles that confer resistance to control efforts should rapidly increase in frequency within *Anopheles* populations in a manner consistent with selective sweeps. When an allele increases in frequency under selection, its linked genetic background comes with it in a process known as genetic hitchhiking. Selective sweeps, through this hitchhiking effect, lead to decreased levels of polymorphism (Smith and Haigh 1974; Kaplan et al. 1989; Stephan et al. 1992), skewed allele frequency spectra (Tajima 1989; Fay and Wu 2000), and increases in linkage disequilibrium surrounding the site under selection (Kim and Nielsen 2004). Classically, methods for finding sweeps have focused on a particular aspect of genetic variation, for instance observing the site frequency spectrum at a locus and comparing it to expectations under neutrality and selective sweeps (Nielsen et al. 2005). More recently, the field has made excellent progress in combining signals across multiple features of genetic variation through supervised machine learning (SML) (Pavlidis et al. 2010; Lin et al. 2011; Ronen et al. 2013; Pybus et al. 2015; Schrider and Kern 2016; Sheehan and Song 2016; Kern and Schrider 2018; Sugden et al. 2018), which has substantially improved power, accuracy, and robustness in what have been stubbornly difficult inference problems within population genetics (Schrider and Kern 2018). Although much attention has been paid to applying SML for the identification and classification of completed selective sweeps in the genome (Schrider and Kern 2016; Kern and Schrider 2018), less effort has been made for using SML to identify sweeps that are incomplete within a population, sometimes called partial sweeps (although see Pybus et al. [2015] and Sugden et al. [2018] for recent examples). In these cases, the beneficial allele is not currently fixed within the population, thereby creating a weaker hitchhiking effect in comparison to a completed sweep, and accordingly a more subtle perturbation of patterns of genetic variation (Coop and Ralph 2012). Although difficult to detect, partial sweeps could be implicated in cases where recently initiated selective forces cause presently ongoing adaptation, directional selection ceases prior to fixation, or an intermediate allele frequency is favored by balancing, polygenic, and/or pleiotropic selection. Therefore, it is important to address the challenge of capturing such genomic signatures that represent a significant facet of evolution, which may also give insight into future dynamics.

## New Approaches

Recent successful efforts to reduce malaria transmission are in danger of collapse due to evolving insecticide resistance (IR) in the mosquito vector *Anopheles gambiae*. We aim to understand the genetic basis of current adaptation to vector control efforts by deploying a novel method that can classify multiple categories of selective sweeps, including partial sweep classes, from population genomic data. To this end, we extend a recent SML method to partial sweep inference and apply it to elucidate ongoing selective sweeps from *Anopheles* population genomic samples.

Specifically, we introduce here an extension of *S/HIC* (Schrider and Kern 2016) and *diploS/HIC* (Kern and Schrider 2018), *partialS/HIC*, which as an SML classifier leverages labeled training examples to learn a mapping from input data space to associated labels. In the case of *partialS/HIC*, data are generated through simulations of genomic segments and converted into summary statistics, similar to *S/HIC* and *diploS/HIC*, to train a deep convolutional neural network (CNN) for classification of a genomic window from a set of selection states that includes both hard and soft partial sweeps along with their associated linked classes (i.e., regions adjacent to either a partial hard or soft sweep). Importantly, this implementation achieves increased inferential power by utilizing dozens of additional summary statistics, including summaries for the distribution of integrated haplotype scores (*iHS*), a summary statistic developed to detect signatures of recent positive selection for an individual single nucleotide polymorphism (SNP) (Voight et al. 2006), as well as derivatives of a recently developed SNP-specific compound statistic called SAFE (selection of allele favored by evolution) (Akbari et al. 2018), under a deep learning framework that involves training CNNs with coalescent simulations that accommodate demographic history and are converted into spatially explicit 2D feature vector images. We validate *partialS/HIC*’s performance through extensive simulation-based experiments that were modeled after data from phase I of the *A. gambiae* 1000 genomes project (Ag1000G) (Miles et al. 2017), with particular emphasis on discovering sweeps currently in progress such as what might result from ongoing vector control efforts (e.g., insecticide spraying). Our findings demonstrate that our method is effective for detecting partial sweeps even in the face of complex population size histories such as those found among the Ag1000G samples. Furthermore, for binary classification of selective sweeps when partial sweeps are included, *partialS/HIC* has greater accuracy than *iHS* or SAFE alone as well as two alternative approaches to sweep inference based on the same suite of summary statistics. Subsequently, we apply our method to the empirical Ag1000G data, revealing many partial sweeps as well as completed sweeps from standing genetic variation. Moreover, we find that our sweep candidates are highly enriched for loci that have been previously identified as contributing to IR.

## Results

### Coalescent Simulations of Feature Vector Images for *partialS/HIC* Training

To train *partialS/HIC*, we deployed the program *discoal* (Kern and Schrider 2016) to perform coalescent simulations of completed and partial as well as hard and soft selective sweeps, along with simulations without sweeps, in a manner analogous to Schrider and Kern (2016) (supplementary fig. S1, Supplementary Material online). Individual simulations were converted into 2D matrices, or feature vector images, built from 89 rows corresponding to different summary statistics, and 11 columns corresponding to adjacent subwindows. The 89 statistics include, along with 14 that were previously implemented in *S/HIC* and/or *diploS/HIC*, 3 genomic region variants of the SNP-specific *iHS* statistic (Voight et al. 2006) and 72 derivatives of the SAFE score (Akbari et al. 2018). *partialS/HIC* is trained to classify genomic segments into one of nine states: unaffected by selection (i.e., neutral); containing a completed hard, completed soft, partial hard, or partial soft sweep, respectively; or linked to a completed hard, completed soft, partial hard, or partial soft sweep, respectively. To this end, we defined the four completed hard/soft and partial hard/soft selective sweep states as containing a sweep within the central, focal subwindow (i.e., the fifth out of the 11 columns of subwindows). In contrast, the four classification states involving linkage to a selective sweep were defined as having a sweep of the given type within one of the remaining ten subwindows. Therefore, every linked selection state was trained from simulations that vary in genetic distance to the sweep target site, such that the total set of simulations all had a sweep occurring in any of the ten nonfocal subwindows. This allowed us to accommodate a range of linked classes that differ in spatial patterns within a genomic window, as well as assess how linkage distance affects misclassification bias during our simulation experiments. Simulations were run for each of eight population size histories corresponding to the empirical Ag1000G population data sets, which were previously inferred as part of the initial data release (Miles et al. 2017). These *Anopheles* population data sets from Miles et al. (2017) are labeled here as AOM (*Anopheles coluzzii* from Angola), BFM (*A. coluzzii* from Burkina Faso), BFS (*A. gambiae* from Burkina Faso), CMS (*A. gambiae* from Cameroon), GAS (*A. gambiae* from Gabon), GNS (*A. gambiae* from Guinea), GWA (*Anopheles* of uncertain species from Guinea-Bissau), and UGS (*A. gambiae* from Uganda).

Heatmaps constructed from median values across simulations reveal expected spatial patterns, such that values immediately flanking a sweep are substantially different than those further from the focal subwindow, whereas neutral regions display no discernible pattern among subwindows (supplementary fig. S2, Supplementary Material online). Additionally, spatial patterns of statistics differ qualitatively between selection states. These observations are consistent regardless of mosquito population history, suggesting that there is signal within this collection of summary statistics to isolate the location of a sweep to a specific subwindow as well as distinguish among neutral regions and types of selective sweeps.

### Deep Learning Excels in Detecting Selective Sweeps, Including Partial Hard Sweeps

**Fig. 1.**
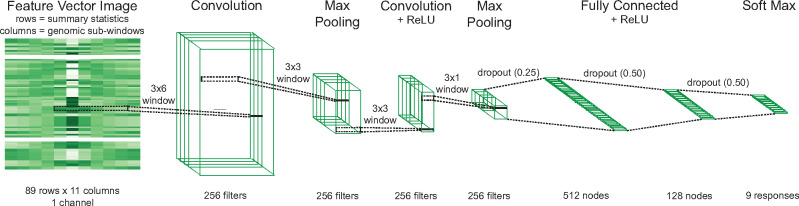
CNN architecture of neural network layers. Our *partialS/HIC* classifier utilizes a convolutional neural network whereby the input feature vector image, composed of 89 summary statistics organized into rows and across 11 contiguous genomic subwindows organized into columns, is passed to a 2D convolutional layer with 256 filters using a 3 × 6 receptive field. Next is a 2D max pooling layer given a 3 × 3 receptive field. Then there is a second 2D convolutional layer of 256 filters based also on a 3 × 3 receptive field and ReLU activation. Afterward is a second 2D max pooling layer with a 3 × 1 receptive field. The tensor is then flattened after a dropout layer (*P* = 0.25) and passed to two fully connected layers with ReLU activation, resulting in 512 and 128 nodes with subsequent dropout (*P* = 0.50), respectively. Finally, a softmax activation layer classifies the nine responses.

**Fig. 2.**
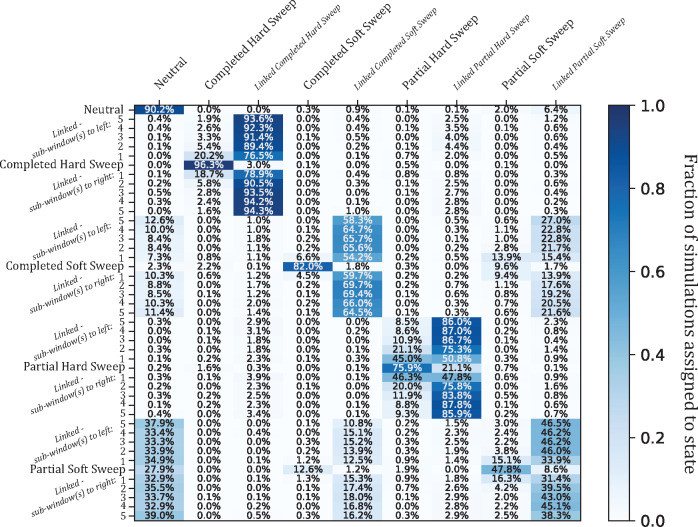
Confusion matrix heatmap of *partialS/HIC* simulation experiment. Given the BFS population history. Each row designates a true simulation class, which for linked sweeps is differentiated by distance in genomic subwindows to the target selective sweep. In total, there are 45 simulated scenarios shown, including 11 for each sweep type (i.e., one case whereby the sweep is within the central subwindow, and 10 whereby a linked sweep is located within one of the flanking subwindows) and neutrality. Each column indicates one of nine inferred states, with the linked simulation classes collapsed into a single category per selective sweep type for training and classification, allowing *partialS/HIC* to learn to distinguish between sweeps located in the focal subwindow and those somewhere nearby. Darker blue cells represent a higher proportion of the 1,000 calls for each true class. Importantly, there is generally high accuracy in discriminating between sweeps within the focal subwindow versus linked subwindows, especially when linkage is beyond one subwindow away and particularly for completed sweeps. However, discovering partial soft sweeps is noticeably a challenging task. Moreover, there is greater sensitivity separating full completion from partial completion for hard sweeps in contrast to soft sweeps.

We utilized *partialS/HIC* to train and test a CNN for nine-state classification independently on each of the eight demographic histories associated with the Ag1000G population samples (supplementary fig. S1, Supplementary Material online and fig. 1). To this end, we produced eight batches of simulations that were split into separate training, validation, and testing sets. During the training process, CNN hyperparameters were tuned on the training set whereas the validation set allowed mitigation of overfitting (supplementary fig. S3, Supplementary Material online). Each CNN was subsequently assessed for accuracy with the corresponding held-out testing set, which was generated under the same specifications as the training/validation data except linked selection classes were kept discrete versus being grouped together into the four linked selection states. Among the eight test sets, there was moderate overall accuracy for this simulation experiment (median accuracy = 66.4%; supplementary table S1, Supplementary Material online). However, confusion matrix heatmaps provide a more informative view of our classifier’s performance, which exhibited reliability in identifying neutral regions, completed hard and soft sweeps, partial hard sweeps, and individual regions linked to completed hard/completed soft/partial hard sweeps (fig. 2 and supplementary fig. S4, Supplementary Material online). In general (i.e., all demographic scenarios save for AOM), completed hard sweep is the class that experienced the highest degree of correct assignment (median accuracy = 96.0%). We also had high accuracy for identification of linked completed hard regions, demonstrating a strong ability to localize completed hard sweeps. The behavior of our *partialS/HIC* classifier is likewise favorable for completed soft sweeps (median accuracy = 84.2%), with completed soft sweeps rarely detected incorrectly as hard sweeps and instead typically mistaken for either the neutral or partial soft sweep state. Moreover, subwindows linked to completed soft sweeps beyond one subwindow away had low levels of misclassification to any of the nonlinked sweep states, again allowing for dependable localization of the sweep.

Importantly, the purpose of *partialS/HIC* is to extend our state space to identify ongoing selective sweeps while distinguishing these from completed sweeps. We find that our ability to identify partial hard sweeps was generally strong across population histories (median accuracy = 74.6%) and is often comparable to that of completed soft sweeps. However, localization of partial hard sweeps along the chromosome was more difficult than for completed sweeps, as can be seen from the moderate levels of confusion between partial hard sweep and linked partial hard sweep subwindows. Undoubtedly, this is due to the limited amount of time recombination has had to whittle down the haplotype carrying the beneficial mutation.

Identifying partial soft sweeps was a much more challenging task (median accuracy = 45.3%), with a high false-negative rate (median rate of misclassification as neutral = 27.6%) as well as a substantial probability of misclassification as a completed soft sweep (median rate = 14.4%). It is encouraging though that partial soft sweeps were almost never misclassified as a completed nor partial hard sweep. Additionally, although our accuracy in classifying partial soft sweeps was poor, false positives were not a major concern (median rate of misclassifying neutral regions as partial soft sweep = 3.2%). Although even fairly low false-positive rates can be problematic when the true number of sweeps is low, *partialS/HIC* achieved acceptably low false discovery rates (FDRs) for partial soft sweeps in our application to the *A. gambiae* 1000 Genomes data set, as we show below. Furthermore, linked partial soft sweeps beyond one subwindow away from the focal subwindow were rarely mistaken for a sweep state, and likewise, partial soft sweeps were seldom confused for being linked (median rate of misclassification as linked partial soft sweep = 4.3%), thus demonstrating that accurate localization of partial soft sweeps may be possible.

In summary, *partialS/HIC* has excellent ability to distinguish partial from completed sweeps for de novo mutations, and lesser yet still substantial power for sweeps from standing variation. Moreover, we demonstrated very strong performance in differentiating between hard and soft sweeps, regardless of whether a sweep was completed or incomplete. Importantly, this is all while maintaining an acceptable false-positive rate across each of the population histories tested (median accuracy for neutral regions = 85.1%; median rate of misclassifying neutral regions as any one of the four nonlinked sweep states = 4.6%).

### Robustness to Demographic Model Misspecification

To assess robustness to demographic misspecification, we applied a CNN trained on simulations from one population sample to data generated from an alternate demographic history. Specifically, we used training data from the GAS population size history, which was fairly stable over time, and leveraged it against the CMS test data set, which experienced a dramatic population expansion (overall accuracy = 55.0%; rate of misclassifying neutral regions as any one of the four nonlinked sweep states = 1.7%). Despite this misspecification, the confusion matrix (supplementary fig. S5, Supplementary Material online) strongly resembles the corresponding matrix that is correctly specified for demography (i.e., for CMS in supplementary fig. S4, Supplementary Material online). In particular, accuracies for finding neutral regions, completed hard sweeps, and partial hard sweeps are roughly equivalent between the correctly specified model and misspecified model (supplementary fig. S5, Supplementary Material online). For soft sweeps, confusion between completed and partial sweeps is increased for the misspecified model, the overall ability to distinguish sweeps from neutrality is largely preserved. Moreover, the rates at which examples from the linked classes were mistaken for sweeps are seemingly unaffected.

To further examine the impact of increasingly misspecified demography, we produced five additional full testing data sets assuming a simple two-epoch instantaneous contraction model. The five test sets differed only by bottleneck severity, which increased in even intervals from 20× to 100×. We then applied the CNN that was trained given the BFS population history and measured overall accuracies. These accuracy measurements are generally similar to that of our baseline, that is, the corresponding original experiment whereby the testing data were simulated under the inferred demography for BFS, thus the demography was correctly specified with respect to the training set underlying this CNN (supplementary fig. S6, Supplementary Material online). However, performance gradually worsens with decreasing bottleneck severity across the test sets, but this may be largely an effect from the number of polymorphisms. To address this, we generated another six test sets that identically replicated the specifications for the BFS training simulations except with *θ* fixed in value yet varying between the six data sets. Notably, the range we utilized here for *θ* exceeded the *θ* prior distribution for the original BFS training and testing simulations, hence further evaluating model misspecification. We find no loss in overall accuracy except in the case with the least genetic diversity simulated, where there is a moderate decrease (supplementary fig. S7, Supplementary Material online). This is unsurprising given that low levels of genetic diversity lead to noisy estimates of the statistics employed by *partialS/HIC*. It is encouraging, however, that sensitivity is maintained throughout most of our *θ* prior distribution range and accuracy does not fall below that of our baseline experiment until *θ* is at a low value toward the bounds (*θ *< 5,000 for the full genomic window size of 55,000 bp). To put this into perspective, genetic diversity measures for the corresponding empirical BFS data set are consistent with simulations generated with *θ *≥ 5,000, and likewise are beyond nearly the entirety of the *θ*  =  1,000 simulated values; among the subwindows that passed our filtering thresholds (supplementary fig. S1, Supplementary Material online), there is a median value of *θ_W_* = 2.633e−2 (central 95% density: 6.484e−3–3.547e−2) and *θ_H_* = 6.318e−3 (central 95% density: 2.381e−3–1.231e−2), compared with a median value of *θ_W_* = 1.218e−2 (central 95% density: 1.116e−3–1.849e−2) and *θ_H_* = 3.863e−3 (central 95% density: 8.592e−4–9.696e−3) across all subwindows generated under the *θ*  =  5,000 condition, whereas the simulations given *θ*  =  1,000 have a median value of *θ_W_* = 2.353e−3 (central 95% density: 9.802e−5–3.861e−3) and *θ_H_* = 7.043e−4 (central 95% density: 5.950e−8–2.571e−3).

Together, these results indicate that sweep discovery and localization is not strongly impacted by several forms of demographic model misspecification during training.

### Partial Sweeps Are Misclassified as Either Completed Sweeps or Neutral at Unpredictable Rates When Not Explicitly Considered

**Fig. 3.**
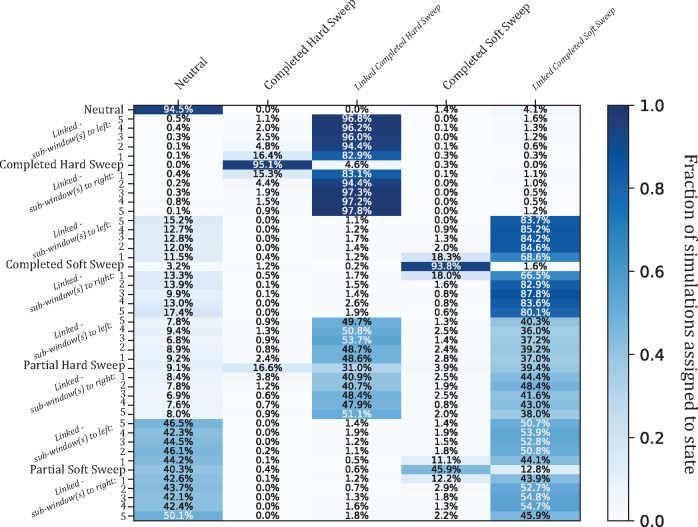
Confusion matrix heatmap of simulation experiment with partial sweeps ignored during training. Given the BFS population history. Structured in the same manner as figure 2, each row designates a true simulation class and darker blue cells represent a higher proportion of the 1,000 calls for each true class. In contrast to figure 2 though, each column indicates one of five inferred states instead of nine, as the two partial sweep and two linked partial sweep classification states were omitted from training to determine misclassification bias when partial sweeps are ignored. There is a substantial decrease in discovery of partial sweeps, especially partial hard sweeps (20.5% total sweep discovery compared with 78.2% in fig. 2; 46.3% total sweep discovery of partial soft sweeps compared with 62.3% in fig. 2). Specifically for partial hard sweeps, a large proportion of the detected sweeps are misclassified as soft sweeps instead of hard sweeps (19.0%; i.e., 3.9% of calls = soft sweeps: 16.6% of calls = hard sweeps).

Since the previous versions of *partialS/HIC* (*S/HIC* and *diploS/HIC*) did not allow for partial sweep selection states, we were interested in how such five-state classifiers would behave when confronted with partial sweeps. To explore this, we conducted a simulation experiment that first removed partial hard and soft sweeps as well as their associated linked classes from the CNN training process, thus training on only five states rather than all nine. Next, in an effort to examine the classification behavior for these five-state CNNs, we applied the full test set that included the partial sweep classes. Unsurprisingly, the trend was for partial sweeps to be most often confused for linked selection (fig. 3 and supplementary fig. S8, Supplementary Material online). Perhaps more concerning is the false-negative rate (i.e., rate at which partial sweeps were misclassified as neutral), which was substantial in partial hard sweeps for several populations (median = 8.8%; max = 32.4%; >1% in all populations) and extreme in partial soft sweeps (>50% in three populations, >40% in three more populations, and >24% in all populations). Partial hard sweeps that were discovered were also often misclassified as a completed soft sweep (median rate of misclassification as completed soft sweep = 5.5%). However, when training included partial sweeps, there is universal and dramatic improvement in both finding sweeps and correctly identifying the model of selection (fig. 2 and supplementary fig. S4, Supplementary Material online). Meanwhile, overall accuracy remains similar among the five-state and nine-state classifiers with respect to simulations of neutral, completed sweep, and linked classes exclusively (fig. 3 and supplementary fig. S8 and table S2, Supplementary Material online). As a result, accuracy should only stand to benefit from incorporating partial sweeps into training since ignoring such information leads to unacceptably high false-negative rates of partial sweeps being called neutral or linked.

### *partialS/HIC* Binary Classification Outperforms Several Competing Approaches

To assess whether the collection of summary statistics under our deep learning method extends inferential resolution beyond the signal conferred by *iHS* or SAFE alone, and to determine if our CNN approach leverages these statistics in a more informative manner than other aggregation schemes, we compared receiver operating characteristic (ROC) curves, which plot true positive against false-positive rates given varying thresholds, for the binary classification task of broadly detecting selective sweeps (i.e., any of the four selection states involving a sweep within the focal subwindow) versus neutral regions or linked sweeps. We selected for our comparison *iHS*-derived statistics (mean, maximum, and proportion of outlier *iHS* values within the central subwindow of the testing simulations) because *iHS* was explicitly designed for detecting partial sweeps (Voight et al. 2006), and SAFE-based statistics (variance and maximum of SAFE scores within the central subwindow of the testing simulations) since SAFE is a compound of many variables, of which the vast majority of our training data are summaries (Akbari et al. 2018). Additionally, we conducted a principal component analysis (PCA) from the training data, restricted to only the focal subwindow as well as for the entire data set throughout the full window, respectively, in two separate PCAs, and projected the testing data onto the first two principal components (PC1, PC2). We then produced an ROC curve from each set of principal component values transformed from the testing data, respectively, to both PC1 and PC2 as well as the PCA on solely the central subwindow versus the full-scale window, for a total of four individual ROC curves. These ROC curves allow contrast to a simple dimensionality reduction approach. Lastly, we computed Composite of Multiple Signals scores from the testing data for both the focal subwindow and full window, respectively, using the training data to derive probability distributions. Deploying the Composite of Multiple Signals metric provides an alternative method that is intended to exploit multiple summary statistics for inference of positive selection (Grossman et al. 2010). For a direct comparison, we altered *partialS/HIC* slightly to create a CNN that had two final output responses (i.e., sweep in central subwindow vs. no sweep in central subwindow) instead of nine, with the training data set as well as architecture of network layers (fig. 1) remaining the same. The CNN optimized for binary classification was then applied to the testing simulations, with the output probability of a selective sweep utilized as input to generate the ROC curve.

**Fig. 4.**
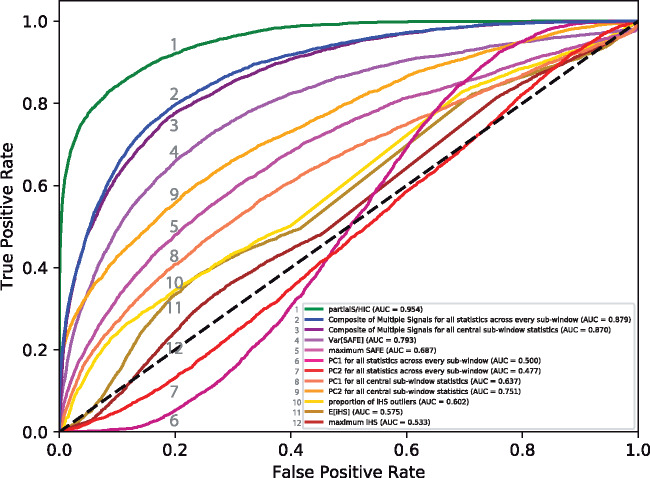
ROC curves for binary classification of selective sweeps, including partial sweeps, versus regions neutrally evolving or under linked selection. Given the BFS population history. Each curve is labeled with a number that is indexed in the legend. The *partialS/HIC* deep learning classifier outcompetes two other approaches for managing the same suite of summary statistics: Composite of Multiple Signals and PCA. Furthermore, *partialS/HIC* excels in performance over several subwindow derivatives of two summary statistics, SAFE and *iHS*, which were all included within our set of summary statistics used for training. Notably, Composite of Multiple Signals, SAFE, and *iHS* were all designed to uncover selective sweeps. Additionally, the SAFE score is itself a compound statistic that captures signal from several constituent statistics, and the majority of our training data is derived from either the SAFE score or one of its components.

The *partialS/HIC* binary classifier consistently outperforms other methods in identifying selective sweeps to the focal subwindow when partial sweeps are included (median AUC = 0.943; fig. 4 and supplementary fig. S9, Supplementary Material online). Interestingly, we demonstrate that the Composite of Multiple Signals also has a strong ability to extract information from our selection of summary statistics, though it always trails behind *partialS/HIC*; in every case, the best performance after *partialS/HIC* is for the Composite of Multiple Signals based on all subwindow statistics (median AUC = 0.876), whereas the next best ROC curve is from the Composite of Multiple Signals calculated from only the central subwindow (median AUC = 0.859). In contrast, with the unexpected exception of PC2 for central subwindow statistics (median AUC = 0.749), PCA appears to be an unfavorable approach for combining summary statistics to infer selective sweeps (e.g., median AUC for PC1 of all subwindow statistics = 0.500). Furthermore, there is evidence that SAFE scores capture important signal about selective sweeps in a manner that is robust to the final frequency of the selected mutation, particularly the variance of SAFE scores within the focal subwindow (median AUC = 0.787). Conversely, there seems to be decreased signal in *iHS*, as all its focal subwindow variants individually performed quite poorly (e.g., median AUC for proportion of *iHS* outliers = 0.582).

### Soft and Partial Sweeps Are Commonplace among *A. gambiae* Populations

**Fig. 5.**
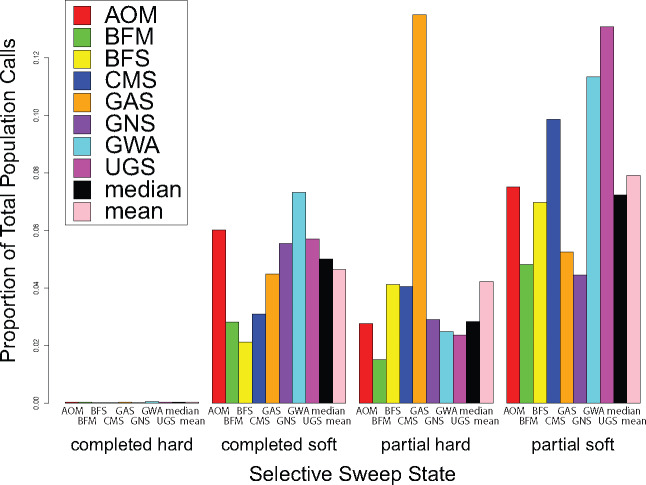
Genome-wide *partialS/HIC* sweep calls across empirical mosquito population data sets. For all eight data sets, there is a minimal number of calls for completed hard sweeps, represented here on the *Y*-axis as the relative proportion to the total set of empirical calls across every genomic window per population data set. However, there is indeed a substantial proportion of sweep calls for hard sweeps, yet are incomplete or in progress. In most cases, the number of both completed and partial soft sweeps further exceeds that of partial hard sweeps. Furthermore, the majority of sweeps have not reach fixation across these *Anopheles* populations.

Turning our attention to the Ag1000G phase I data, we applied our nine-state CNNs to the corresponding *A. gambiae* population data sets, classifying 5 kb segments using a 55 kb full sliding window throughout the whole genome (supplementary fig. S1, Supplementary Material online). Each of the eight mosquito populations contains a large number of subwindows identified as completed soft sweeps (median fraction of total calls genome wide = 5.01%) as well as partial sweeps (median fraction of total calls genome wide for partial hard sweep = 2.84%; median fraction of total calls genome wide for partial soft sweep = 7.24%), coupled with only a handful of completed hard sweep predictions (median fraction of total calls genome wide = 0.03%) (fig. 5 andsupplementary table S3, Supplementary Material online). Partial soft sweeps were typically discovered most often (median proportion of sweep calls = 52.59%), with completed soft sweeps often following (median proportion of sweep calls = 28.80%) and partial hard sweeps usually being the third most numerous class of detected sweep (median proportion of sweep calls = 19.75%). Notably, our estimated FDRs are higher for soft sweeps (median FDR for completed soft sweeps = 11.09%; median FDR for partial soft sweeps = 12.20%) compared with hard sweeps (median FDR for completed hard sweeps = 0.00%; median FDR for partial hard sweeps = 0.39%); this implies that individual soft sweep candidates should be viewed with more caution, though we should be able to estimate the genome-wide proportion of these classes well. Specifically, classifications for partial soft sweeps outnumber those for completed soft sweeps in every population besides GNS, as well as partial hard sweep calls in all populations but GAS. Importantly, these findings remain the same, with the exception that calls for partial hard sweeps now slightly exceed partial soft sweep classifications in GNS, after false discovery correction (supplementary table S3, Supplementary Material online). Additionally, had partial sweeps not been accounted for in the training process, our results suggest that we would have both underestimated the total number of sweeps and incorrectly labeled many of our partial sweeps (fig. 3 and supplementary fig. S8, Supplementary Material online). This would have led to the conclusion that adaptation from standing variation rather than de novo mutations dominates selective sweep dynamics in these *A. gambiae* populations. Although it is clear that soft sweeps are indeed more common in these data, our results suggest that hard sweeps often occur as well, though with few reaching fixation. Furthermore, the *partialS/HIC* classifications indicate that most selective sweeps in these population samples are incomplete, suggesting that we are capturing a view of selection in progress. Notably, these results are conditioned on training simulations that assumed each population as independent with no admixture, which may have in fact been significant in certain cases. However, since our simulation experiments demonstrate that *partialS/HIC* is robust to demographic misspecification, we believe this may be a minor concern.

### Selective Sweeps Are Significantly Enriched in Functional Regions of the *A. gambiae* Genome

**Fig. 6.**
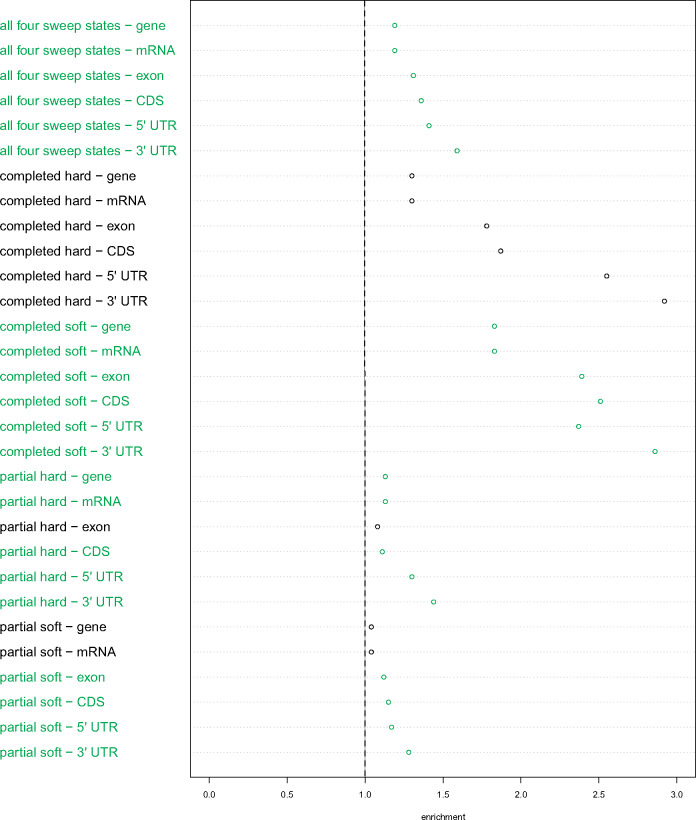
Enrichments of *partialS/HIC* empirical selective sweep calls within DNA regions. For the BFS population empirical data set. The vertical dotted line represents the threshold for enrichment (i.e., values exactly along the dotted line indicate single-fold enrichment) based on the permuted calls, with rows that are statistically significant (*P *<* *0.05) in green. Completed soft sweeps are enriched within all six DNA regions that experienced permutation tests. Similarly, all of these DNA regions are enriched with partial sweeps, either both hard and soft or only one of the two cases (though this is not true for all populations; supplementary fig. S10, Supplementary Material online). In contrast, there is no significant enrichment of completed hard sweeps for any of the DNA regions.

To elucidate broad characteristics underlying the genomic targets of selection, we used permutation tests of sweep call locations to discover enrichment patterns in the following DNA regions of interest: gene, mRNA, exon, coding sequences (CDS), five-prime UTR, and three-prime UTR. Permutation tests were based on the total number of calls for the four selection states with sweeps occurring within the focal subwindow, as well as the individual number of calls for each of these states, respectively. Across all eight population data sets and for all six DNA regions under investigation, there is a statistically significant enrichment of total sweep calls along with completed soft sweeps calls in particular, whereas completed hard sweep calls are not significantly enriched in any single case (fig. 6 and supplementary fig. S10 and table S4, Supplementary Material online). Conversely, partial sweep enrichment varies among populations as well as individual DNA regions. Specifically, partial hard sweeps are significantly enriched in five of the six DNA regions for BFS and CMS, and four of the DNA regions for UGS; whereas partial soft sweeps are significantly enriched in all six DNA regions for UGS, five of the DNA regions for BFM, and four of the DNA regions for BFS.

### IR Loci, Especially Related to Metabolism, Are Significantly Enriched for Selective Sweeps

**Fig. 7.**
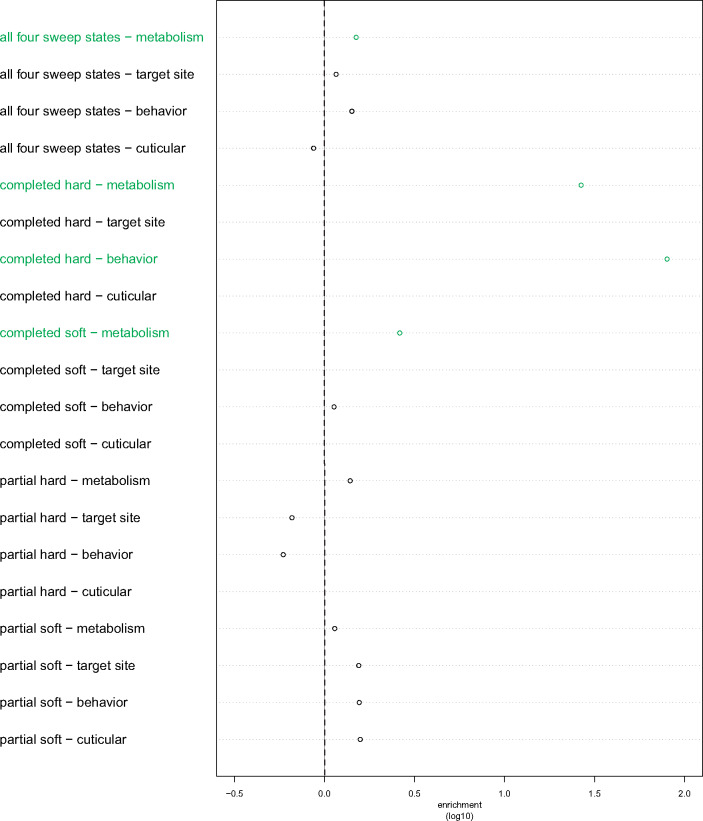
Enrichments of *partialS/HIC* empirical selective sweep calls within groupings of IR genes. For the BFS population empirical data set. The vertical dotted line represents the threshold for enrichment (i.e., values exactly along the dotted line indicate single-fold enrichment) based on the permuted calls, with rows that are statistically significant (*P *<* *0.05) in green and rows without a plotted point resulting from the IR gene group not having any calls of that sweep type (e.g., there are no completed hard sweeps called within target site genes). In this mosquito population, there is evidence for several mechanisms through which IR evolved, both in sweep types (i.e., completed hard as well as completed soft sweeps) and categories of genes under selection (i.e., metabolism and behavior). Notably, partial sweeps are not inferred to be enriched during the evolution of IR genes here, though they are for other populations (supplementary fig. S11, Supplementary Material online).

We performed a similar permutation analysis for four sets of genes known to confer IR, finding at least one set of IR genes to be statistically significant for every population data set in enrichment of total sweep calls (i.e., aggregate of all four sweep classes) and completed soft sweeps, respectively (fig. 7 and supplementary fig. S11 and table S5, Supplementary Material online). In particular, metabolism-related IR genes are significantly enriched for each of these cases. Furthermore, IR genes corresponding to well-characterized resistance loci (i.e., target sites) are significantly enriched for AOM and BFM total sweep calls as well as completed soft sweeps in AOM, BFM, and GNS; IR genes associated with behavior are significantly enriched for BFM and GNS total sweep calls as well as completed soft sweeps in AOM, CMS, and GAS; and IR genes affiliated with cuticular activity are significantly enriched for completed soft sweeps in GWA and UGS. In contrast, completed hard sweeps are only significantly enriched in BFS, GNS, GWA, and UGS for IR genes connected to behavior (as well as metabolism for BFS). For partial sweeps, significant enrichment only occurs within BFM (partial soft sweeps in metabolism as well as behavior IR genes), CMS (partial hard sweeps in metabolism as well as target site IR genes), and UGS (partial hard sweeps in metabolism IR genes).

### Completed Soft Sweeps Are Significantly Enriched within the Same Gene Ontology Term Annotations across Populations

Beyond IR, we were interested in asking what sorts of functional annotations, if any, were enriched in our sweep candidates. Toward this end, we again used a permutation approach, asking for evidence of individual gene ontology (GO) term enrichment. We used the same protocol as with the IR loci, except with an FDR correction appropriate for the GO sites. We found that partial soft sweeps are not significantly enriched for any GO terms among populations, whereas completed hard sweeps and partial hard sweeps are each enriched only in UGS for a single GO term. However, for completed soft sweeps, we inferred many statistically significant enrichments, several of which were widely shared across populations (supplementary table S6, Supplementary Material online). Specifically, there are six cases of the same GO term in all eight populations; three of these are related to cellular components (“nucleus,” “membrane,” and “integral component of membrane”), and the other three are connected to molecular function, specifically binding (“nucleic acid binding,” “protein binding,” and “ATP binding”). Notably, one of these GO terms (“membrane”) is likewise the single example for which completed hard sweeps are enriched, within UGS. Other cases involving the same GO term significantly enriched for completed soft sweeps in over half of the populations include: “binding,” “cytoplasm,” and “zinc ion binding” in seven populations; “RNA binding” in six populations; and “mRNA splicing, via spliceosome” and “ATPase activity” in five populations. These results suggest that beyond IR, selection on standing variants may have occurred in parallel across regions and/or with widespread geographic impact so as to affect multiple populations simultaneously.

## Discussion

### *partialS/HIC* Elucidates Both Species-Wide and Population-Specific Sweep Dynamics within *A. gambiae*

The Ag1000G data provided the opportunity to investigate selection at both the continental scale, where wide-reaching impact across the whole species complex could be uncovered, and the regional level, revealing population-specific sweep dynamics. For the former, we observed that *A. gambiae* populations consistently experienced very few completed hard sweeps, with nearly all sweeps being partial and/or soft. In fact, the impact of completed hard sweeps on the adaptive process within mosquitos appears to be even more limited than what was observed previously in humans (Schrider and Kern 2017). This is likely a result of the much larger population sizes and concordant levels of genetic variation that are maintained within *Anopheles* populations. Importantly, we find a large number of ongoing selective sweeps within these populations, particularly in comparison to the number of completed sweeps. There are multiple reasons why this might be the case. A trivial explanation may simply be that we only have power to detect sweeps that have completed in the past few hundred generations, though this seems unlikely. More plausibly, a large number of ongoing sweeps might be expected given the recent change in environment induced by vector control efforts. Another possible explanation is that the frequency dynamics of beneficial alleles within a population is often more complex than assumed and may indeed contain an overdominant component (Sellis et al. 2011). This would mean that some portion of the partial sweeps that we are observing in *Anopheles* is actually balanced, or transiently balanced, polymorphisms. A fourth class of explanation is that beneficial mutations may not be able to fix in populations due to competition with beneficial mutations on other genes that have originated in different parts of the species range (Ralph and Coop 2010). Indeed, each of these factors may play some role in our reported abundance of partial sweeps.

Although such genome-wide sweep patterns occur species-wide, enrichment behavior seems much more population-specific. For instance, although every population possesses significant enrichment of completed soft sweeps coupled with no completed hard sweep significant enrichments for the six functional DNA regions studied here, partial sweep enrichments vary widely among data sets. Sweep behavior is even more idiosyncratic for IR genes, as the only constant between populations is that metabolism is a recurring target of selection, especially for completed soft sweeps.

These findings from the Ag1000G data provide important genomic resources that could inform continental-wide malaria control strategies for the entire *A. gambiae* species complex, as well as have relevance to management efforts specialized to certain populations and localities. Such insight into mosquito vector evolution may also help curb future IR adaptation, and in turn prevent impending crises of vector control failure. However, it is important to consider that our partial sweep calls could be capturing more complex selective dynamics at play, for example, polygenic and quantitative trait adaptation (Pritchard et al. 2010; Booker et al. 2017), balancing selection (Connallon and Clark 2013), and introgression of beneficial alleles. These could lead to different modes of adaptation for the same genomic region across populations, for instance, a favorable SNP undergoing a soft sweep at its origin and then carried to neighboring populations (as was suggested in Miles et al. [2017]) may appear to be experiencing a partial hard sweep in those recipient populations. Such complicated interactions merit further investigation on the Ag1000G data, which would not only continue advancing methodological development for population genetics, but also address interesting questions for a widespread and ecologically important organism that has crucial ramifications on wildlife management and public health.

### *partialS/HIC* Offers Unprecedented Detection of Partial Sweeps

SML approaches are rapidly gaining traction among population geneticists, with deep learning in particular beginning to experience increased attention and methodological development due to its exciting potential to unlock classic population genetics problems. Examples of successful SML implementation in population genomics include demographic model choice (Smith et al. 2017), demographic parameter inference (Pudlo et al. 2016), comparative analysis of independent single-population size changes (Xue and Hickerson 2017), identification of introgressed regions (Schrider et al. 2018), recombination rate estimation (Lin et al. 2013; Adrian et al. 2016; Gao et al. 2016), and genomic scans of selective sweeps (Schrider and Kern 2016); deep learning specifically has been employed for joint inference of demography and selection (Sheehan and Song 2016), discovery of recombination hotspots (Chan et al. 2018), estimation of demographic and recombination parameters (Flagel et al. 2019; Adrion et al. 2020), discovery of functional variants (Zhou and Troyanskaya 2015), prediction of geographic origin (Battey et al. 2020), and differentiating between hard and soft sweeps from neutral regions (Kern and Schrider 2018). These applications especially benefit from the ability to handle high-dimensional input data and bypassing the need of a likelihood function, which is due to SML uncovering data patterns from leveraging a priori information through a training algorithm (Sheehan and Song 2016,Schrider and Kern 2018). CNNs expand this utility to image processing, which has been demonstrated with *diploS/HIC* to be a powerful tool for exploiting the genomic spatial distribution of multiple population-level summary statistics to detect selective sweeps (Kern and Schrider 2018).

Here, we demonstrated with *partialS/HIC* that deep learning can be extended to partial sweeps, especially partial hard sweeps, yielding greater accuracy and robustness than has been previously attained. We also showcased consistent performance in the face of several underlying demographic backgrounds, including when the demography is drastically misspecified. Specifically, *partialS/HIC* is capable of discovering selective sweeps when partial sweeps are considered, simultaneous disambiguation between partial and completed sweeps as well as between hard and soft sweeps, and spatial localization of selection targets in the genome. Moreover, we have shown that partial sweeps remain mostly undetected if ignored from the training process, even though such selection may be commonplace throughout a genome as with the Ag1000G data. As a result, many previous studies scanning for either complete or ongoing selective sweeps solely (i.e., not jointly inferring both types of selection) may have overlooked an important subset of evolutionary events (Ralph and Coop 2010). Researchers may then be interested in re-examining data sets with *partialS/HIC* to elucidate the relative contributions of fixed versus incomplete sweeps to adaptive evolution.

Importantly, the efficacy of *partialS/HIC* relies on several factors that are unexplored here, including simulation prior specifications, CNN architecture with respect to construction and parameterization of neural network layers, and data structure. Hence, it is prudent for future implementations to validate performance by testing a range of configurations, given a project’s individual intricacies, to assess robustness and inherent assumptions. In particular, future exploration of alternate image constructions could be potentially of great methodological benefit. Such images could be derived from different ordering schemes and/or suites of summary statistics, as well as without summary statistics entirely, instead directly exploiting sequence alignments (Chan et al. 2018; Battey et al. 2020; Adrion et al. 2020) or even raw reads. More broadly, CNNs can be further extended to address other long-standing efforts in evolutionary biology, such as parameter inference under complex isolation-migration models or phylogenetic reconstruction (Suvorov et al. 2020).

## Materials and Methods

### Simulations for Training and Testing CNN Classifier

We used *discoal* (Kern and Schrider, 2016) to simulate training and test data sets corresponding to each *A. gambiae* population under nine different selection states: neutrally evolving, completed hard sweep, completed soft sweep, partial hard sweep, partial soft sweep, and linked region for every one of the four sweep classes (supplementary fig. S1 and table S7, Supplementary Material online). For the four sweep types, the target SNP was located in the exact middle position within the central, or sixth in sequence, subwindow of 11 in total; the selected SNP was placed in the middle within one of the other ten subwindows for linked sweeps. There were 2,000 training examples per selection state (with the specific subwindow under selection, i.e., one of ten possible simulation classes, randomized for linked sweeps) and 1,000 test examples per class (including for each of the ten linked sweep locations), resulting in a training data set of 18,000 simulations and a test data set of 45,000 simulations given each demographic history, thus totaling 144,000 training and 360,000 test simulations (as well as 495,000 additional test simulations from 11 test data sets all under the BFS population history to explore demographic misspecification; see below). To conduct single-population simulations with *discoal*, we used the *stairway plot* (Liu and Fu 2015) point estimates from Miles et al. (2017) for size change parameters as well as *N*_0_ (present-day effective population size), assumed a mutation rate (*μ*) of 3.5 × 10^−9^ mutations per base pair per generation, and performed random draws for locus-wide mutation and recombination rates from the following distributions for each independent replicate: *θ* ∼ *U*($\frac{2\;\times \;E[\theta ]}{11.0}$, $\frac{2\;\times \;E[\theta ]}{1.1}$), whereby *E*[*θ*] = 4 *N*_0_*μL* and *L* is the length of the simulated sequence with *L *=* *55,000 bp; *ρ* ∼ *TEXP*(2 × *E*[*θ*], 6 × *E*[*θ*]), where *ρ*  =  4 *N*_0_*rL*, *r* is the recombination rate per base pair, and *TEXP*(*β*, maximum value) is a truncated exponential distribution with mean *β*; *s* ∼ *U*(1.0 × 10^−4^, 1.0 × 10^−2^); end time of sweep ∼ *U*(0, 2,000) generations ago, which represents fixation for completed sweeps and the transition back to neutral evolution for partial sweeps; selected SNP allele frequency at onset of soft sweep ∼*U*($\frac{1}{N_{0}}$, 0.2); and selected SNP allele frequency at end of partial sweep ∼ *U*(0.20, 0.99).

### Constructing 2D Feature Vector Images of Summary Statistics

The eight training and test data sets (including an additional 11 BFS data sets for testing demographic misspecification), as well as empirical data sets, were converted into 2D feature vector matrices for downstream deep learning (supplementary fig. S1, Supplementary Material online); this was performed within the Python environment and required usage of the module *numpy*. Prior to this 2D transformation, the simulated data were modified to better account for uncertainty within the empirical data, specifically: 1) sites that were missing any individual calls or could not be polarized against the outgroup were excluded; and 2) incorrect identification of the derived allele. For the former, each simulation randomly drew from a distribution of 1,552 masking profiles (with test simulations drawing without replacement per selection class of 1,000 simulations), which determined the exact sites to be omitted from further analysis; a masking profile consisted of the site positions within a single full 55 kb window on the *A. gambiae* genome that had absent at least one sample throughout the entirety of the Ag1000G data and/or ancestral state information, and the total set represented all 1,552 sequential, nonoverlapping windows (e.g., 2 L: 1–55,000; 2 L: 55,001–110,000; 2 L: 110,001–165,000, etc.) where the proportion of masked sites did not exceed 75% in any of the constituent subwindows (i.e., 1,250 sites). To account for mispolarization, estimated rates were obtained from Miles et al. (2017) and exploited via a binomial distribution to mispolarize a random subset of SNPs to the other allele per simulation.

The empirical data similarly underwent processing for compatibility with the simulated data. First, chromosomes were delineated into sequential 5 kb subwindows (e.g., positions 1–5,000 formed the first subwindow, positions 5,001–10,000 formed the second subwindow, etc.), with the aforementioned masking criteria applied across sites and remaining SNPs polarized. Within each population data set, all polymorphic positions composed of more than two alleles were further removed from analysis, such that only polarized monomorphic and biallelic sites comprising a full data matrix of no missing data were left. Subwindows containing no SNPs or less than 25% of the original sites were subsequently discarded, and every configuration of 11 contiguous 5 kb subwindows of those remaining formed a single full window, which would be classified into one of the nine selection states based upon its central subwindow while using spatial information from the neighboring five subwindows on either side. To clarify, this eliminated any window that did not have a consecutive sequence of 11 subwindows that survived data filtering, and resulted in a sliding window that progressed a single subwindow at a time, such that succeeding full windows could be overlapping by up to ten subwindows.

Every independent simulation totaling 55 kb in length from 11 subwindows of 5 kb, as well as empirical sequence of 11 adjacent subwindows per population, was then transformed into 89 separate summary statistic vectors that capture aspects of population-level variation across the sampled individuals, with each vector consisting of 11 elements corresponding to the subwindows. The first 17 summary statistics were *π* (Tajima 1983), *θ_W_* (Watterson 1975), Tajima’s *D* (Tajima 1989), *θ_H_* (Fay and Wu 2000), Fay-Wu’s *H* (Fay and Wu 2000), number of unique haplotypes, *H*_1_ (Garud et al. 2015), *H*_12_ (Garud et al. 2015), *H*_2_/*H*_1_ (Garud et al. 2015), *Z_nS_* (Kelly 1997), maximum *ω* (Kim and Nielsen 2004), *E*[*iHS*) (Voight et al. 2006), maximum *iHS* (Voight et al. 2006), proportion of outlier *iHS* values (Voight et al. 2006), variance of pairwise genotype distances (Kern and Schrider 2018), skewness of pairwise genotype distances (Kern and Schrider 2018), and kurtosis of pairwise genotype distances (Kern and Schrider 2018). These were previously implemented in *S/HIC* (Schrider and Kern 2016) and/or *diploS/HIC* (Kern and Schrider 2018) except for *H*_1_ (though a multilocus genotype equivalent was used in *diploS/HIC*) and the *iHS*-based statistics. We employed the Python package *scikit-allel* to calculate *π*, *θ_W_*, Tajima’s *D*, *θ_H_*, Fay-Wu’s *H*, number of unique haplotypes, *H*_1_, *H*_12_, *H*_2_/*H*_1_, and the statistics related to *iHS*. Values for *iHS* were standardized within 50 derived allele frequency bins, following mispolarization in the case of simulated data. Outlier *iHS* values were defined as within either 2.0% tail of the distribution obtained from simulations of neutral evolution under the appropriate demographic history.

The remaining 72 summary statistics were distribution summaries of SAFE and its various components (Akbari et al. 2018), specifically: haplotype allele frequency (HAF), which is the sum of derived allele counts across all the derived alleles present within a sequence; unique HAF score (i.e., each unique HAF value is counted only once, even if representing multiple individuals); *φ*, which is the sum of HAF scores for sequences harboring the derived allele, divided by the total sum of HAF scores across all sequences; κ, which is the proportion of distinct HAF scores that carry the derived allele; derived allele frequency; and the SAFE score itself, which is the difference between *φ* and κ normalized against the derived allele frequency. Notably, HAF is calculated per sequence, whereas *φ*, κ, derived allele frequency, and SAFE are calculated per polymorphism. The following distribution summaries were utilized to construct individual values spanning a subwindow: mean, median, mode; 2.5%, 25%, 75%, and 97.5% quartiles; maximum, variance, standard deviation, skewness, and kurtosis. Importantly, each summary statistic vector was normalized, in the same manner as the preceding versions to *partialS/HIC* (Schrider and Kern 2016; Kern and Schrider 2018), to capture signal solely from the relative spatial distribution of the summary statistics across the 11 subwindows, rather than allowing influence from absolute values. Subsequently, the 89 vectors were vertically collated to form a 2D matrix that could then be exploited for image processing. The arrangement of these vectors was such that the 11 columns corresponded to the series of subwindows from left to right, and the 89 rows of summary statistics were in the order presented here (with the distribution summaries iterating first for every SAFE component, e.g., row 52: skewness of *φ* values; row 53: kurtosis of *φ* values; row 54: *E*[κ]). Importantly, column and row order affects deep learning optimization, which may have consequences on overall efficacy, due to the convolutional and pooling windows employed by the CNN architecture, hence related summary statistics were grouped together (e.g., alternative distribution summaries of an SNP-based statistic, various SAFE derivatives). Heatmap images, based on median values per statistic and subwindow, were generated in R for the neutral case and each of the four sweep states under every population history from the training simulations.

### Training and Testing CNNs for Deep Learning Implementation

The architecture of our CNN was composed of the following sequential layers: 1) 2D convolutional layer with 256 filters using 3 × 6 windows and “same” padding; 2) 2D max pooling layer given a 3 × 3 window; 3) a second 2D convolutional layer of 256 filters based also on 3 × 3 windows, “same” padding, and ReLU activation; 4) a second 2 D max pooling layer with a 3 × 1 window; 5) dropout layer with *P* = 0.25; 6) flattening layer; 7) fully connected layer with ReLU activation to 512 responses; 8) a second dropout layer with *P* = 0.50; 9) a second fully connected layer with ReLU activation to 128 elements; 10) another dropout layer with *P* = 0.50; and 11) softmax activation layer to 9 states (fig. 1). This architecture was trained using the Python module *Keras* (Chollet 2015) given the *Adam* optimizer (Kingma and Ba 2014), with 20 epochs, batch size of 32 simulations per step within an epoch, and 10% of the training data (e.g., 1,800 simulations from the total nine-state training data set) randomly removed as a validation set during optimization (supplementary fig. S1, Supplementary Material online). Training was performed for every population demography under three experimental settings: 1) given the full set of training data distributed across nine selection states; 2) exploiting a subset of the training data from only five of the selection states, specifically those involving neutral regions or completed sweeps; 3) deploying the entire training data, but with binary classification between selective sweeps in the focal subwindow and all unselected classes (i.e., neutral class together with every linked class). The three training regimes were then applied to the test data set that corresponded in underlying simulated history, resulting in predictions of selection state given the default *Keras* threshold parameters for the first two training schemes, whereas the softmax probability of a focal sweep was instead exploited under training on binary classification. In the former case, individual inferences per test simulation were collated and compared against true values to produce overall accuracy measures as well as confusion matrix heatmaps to assess misclassification bias for each of the 45 simulated scenarios (i.e., 11 subwindow sweep locations for each of the four sweep types, plus neutrality).

Moreover, to explore the effect of demographic misspecification, we conducted a single additional test under the first experimental set-up whereby the CNN trained on the GAS simulations was applied to the CMS test simulations. Furthermore, we engaged in two more misspecification experiments that involved simulating, for the first experiment, five testing data sets that changed the underlying demographic model, and for the second experiment, six testing data sets that differed in fixed *θ* value. In the five test sets that altered the demographic history, we used an instantaneous contraction model that experienced a population crash at 0.0001 time units with an intensity of, respectively, across the five data sets: 20×, 40×, 60×, 80×, 100×. Priors for *θ*, *ρ*, *s*, end time of sweep, selected SNP allele frequency at onset of soft sweep, and selected SNP allele frequency at end of partial sweep remained the same as for the initial BFS simulations. In the six test sets that varied *θ*, we employed the same parameterization from the initial BFS simulations with the exception that *θ* was set to, respectively, across the six data sets: 1,000; 5,000; 10,000; 15,000; 20,000; 25,000. Our motivation for these intervals was to exceed the bounds of the original prior distribution, *θ* ∼ *U*(1,750.204699, 17,502.046985). After simulation, we leveraged the nine-state CNN trained under the BFS specifications against each of the 11 testing data sets to obtain overall accuracies for comparison among these test data sets and to the original test data set whereby the BFS history was correctly modeled.

Regarding the binary classification experiment, the Python module *sklearn* (Pedregosa et al. 2011) was used for building the ROC curves to evaluate accuracy and sensitivity. Additionally, *sklearn* was utilized for conducting PCA on the training simulations, on either the focal subwindow or across the entire full-scale genomic window, respectively, with subsequent projection of the testing simulations into independent sets of PC1 and PC2 values, to obtain a total of four individual ROC curves. To calculate the Composite of Multiple Signals (Grossman et al. 2010), training data were separated into the two categories of selection versus neutrality (i.e., 4,000 simulations from four selection states versus 41,000 simulations from 41 neutral or linked classes), which were, respectively, converted into a histogram of 200 bins for every summary statistic. Assuming the Composite of Multiple Signals for the central subwindow only, this resulted in a total of 178 distributions given the 89 statistics for both selection and neutral; for all subwindows, this was instead a total of 1,958 distributions due to the 11 total subwindows of statistics. These distributions were then employed to calculate probabilities both under selection and neutrality for the corresponding testing data summary statistics; for bins with a zero value, 0.1 of the smallest possible increment (i.e., selection: $\frac{0.1\;\times \;1}{4,000}$; neutrality: $\frac{0.1\;\times \;1}{41,000}$) was assigned as the probability. To determine the prior for each probability, represented in the original Composite of Multiple Signals equation by π, we utilized the proportional composition of the training simulations (i.e., selection: $\frac{4}{45}$; neutrality: $\frac{41}{45}$). Finally, probabilities were natural log-transformed and summed among summary statistics to compute the Composite of Multiple Signals.

### Detecting Selective Sweeps for *A. gambiae* Population Data sets

To scan the genome for signatures of selective sweeps, the nine-state trained CNNs were applied to the eight empirical mosquito data sets, with the underlying simulated demography matched to the sampled population (supplementary fig. S1, Supplementary Material online). Calls were corrected for false discovery by exploiting the accuracy and error rates for neutral regions from the nine-state simulation experiment, such that the amount of neutral calls was assumed to be underestimated whereas the amount of calls for the remaining eight selection states were assumed to be inflated. Subsequently, we produced sets of 10,000 randomly permuted calls across the genome to derive null expectations of sweep enrichment, following Schrider and Kern (2017). Using the gene annotation file “Anopheles-gambiae-PEST_BASEFEATURES_AgamP4.7.gff3.gz” from VectorBase, we exploited these permuted data sets to assess statistically significant enrichment within certain DNA regions, groupings of known IR genes (N. Harding, *pers. comm.*), and all basic GO term definitions from http://www.geneontology.org (last accessed February 18, 2015). The DNA regions of interest included gene, mRNA, exon, CDS, five-prime UTR, and three-prime UTR; IR genes were assigned to four functional categories: metabolism, target sites, behavior, and cuticular. To determine significant enrichment, the number of inferred calls for a particular DNA region or IR gene category had to have a *P* value < 0.05 based on the respective distribution of 10,000 permutations; for the GO terms, we deployed a corrected *q*-value <0.05 due to concerns of false discovery stemming from the large number of terms tested for enrichment.

## Supplementary Material

Supplementary data are available at *Molecular Biology and Evolution* online.

## Supplementary Material

msaa259_Supplementary_Data
