# Supplementary material for: Discovery of Ongoing Selective Sweeps within Anopheles Mosquito Populations Using Deep Learning
Source: Mol Biol Evol. 2020 Oct 6;38(3):1168–83. doi: 10.1093/molbev/msaa259 (PMC7947845; doi:10.1093/molbev/msaa259)
Supplement: msaa259_Supplementary_Data [file msaa259_supplementary_data.zip › Supplemental Figures.pdf]

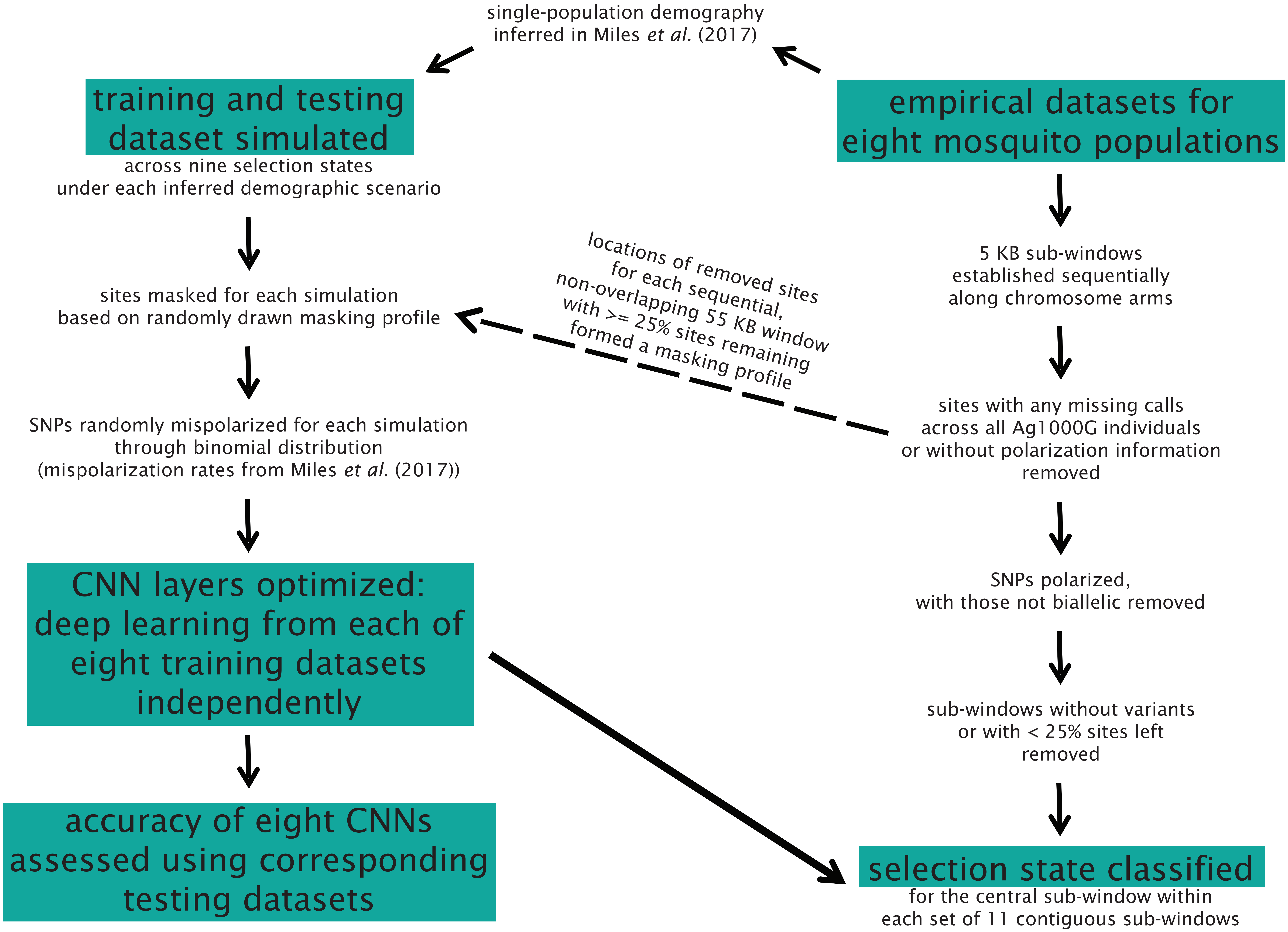
single-population demography inferred in Miles *et al.* (2017)
training and testing dataset simulated
across nine selection states under each inferred demographic scenario
empirical datasets for eight mosquito populations
5 KB sub-windows established sequentially along chromosome arms
locations of removed sites for each sequential, non-overlapping 55 KB window with >= 25% sites remaining formed a masking profile
sites masked for each simulation based on randomly drawn masking profile
sites with any missing calls across all Ag1000G individuals or without polarization information removed
SNPs randomly mispolarized for each simulation through binomial distribution (mispolarization rates from Miles *et al.* (2017))
SNPs polarized, with those not biallelic removed
CNN layers optimized: deep learning from each of eight training datasets independently
sub-windows without variants or with < 25% sites left removed
accuracy of eight CNNs assessed using corresponding testing datasets
selection state classified
for the central sub-window within each set of 11 contiguous sub-windows

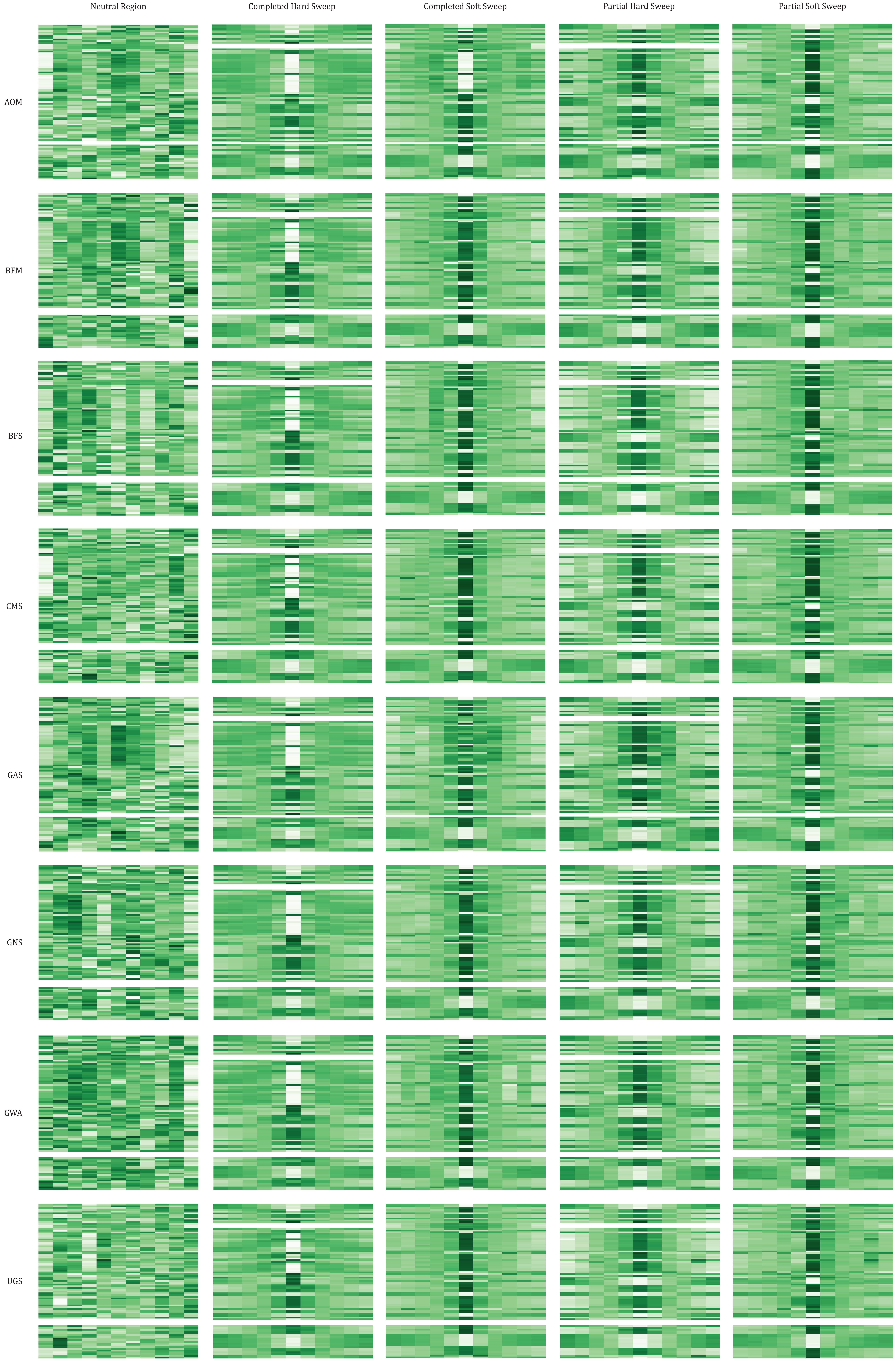
Neutral Region
Completed Hard Sweep
Completed Soft Sweep
Partial Hard Sweep
Partial Soft Sweep
AOM
BFM
BFS
CMS
GAS
GNS
GWA
UGS

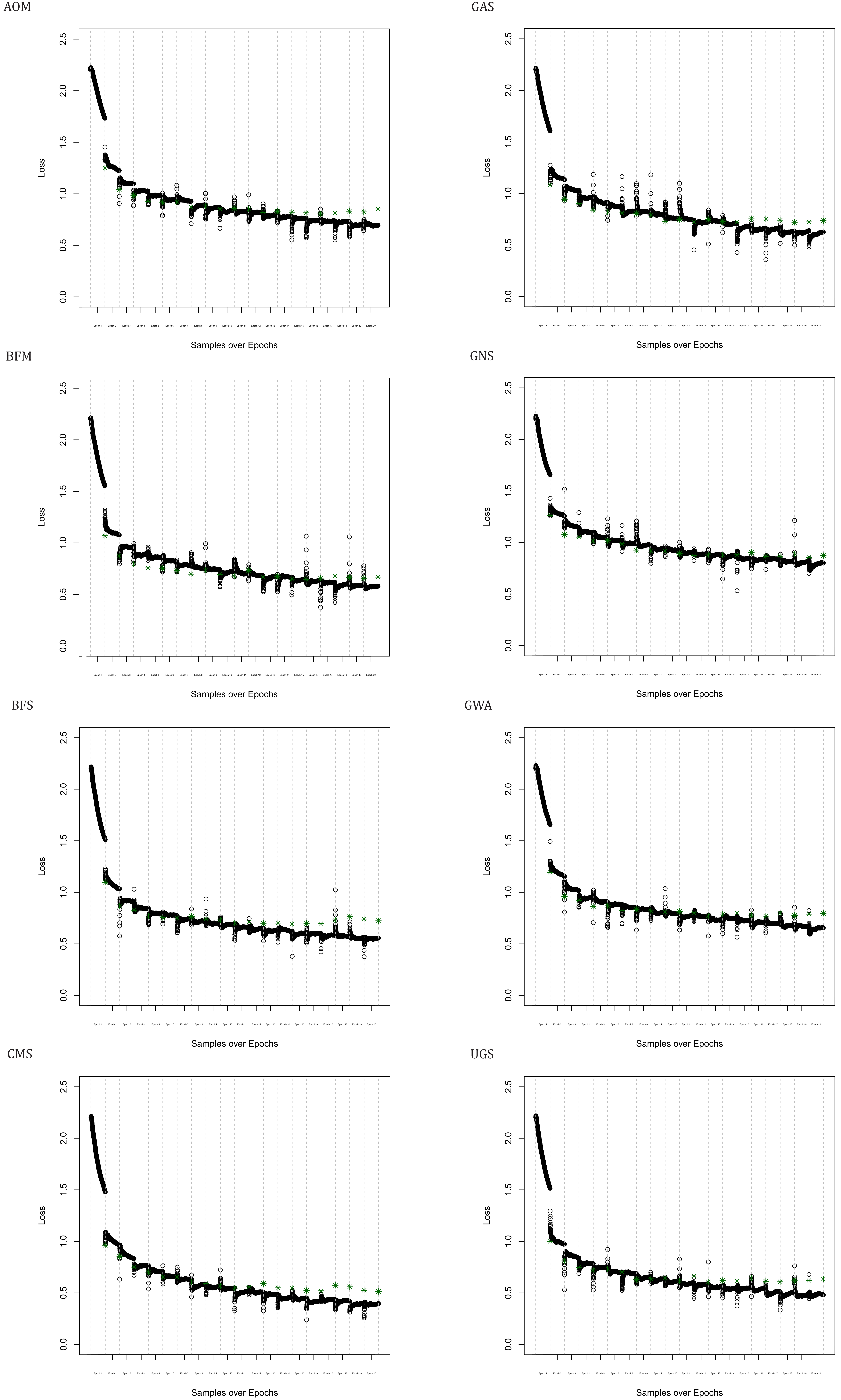

AOM
GAS
BFM
GNS
BFS
GWA
CMS
UGS
Loss
Samples over Epochs
0.0
0.5
1.0
1.5
2.0
2.5

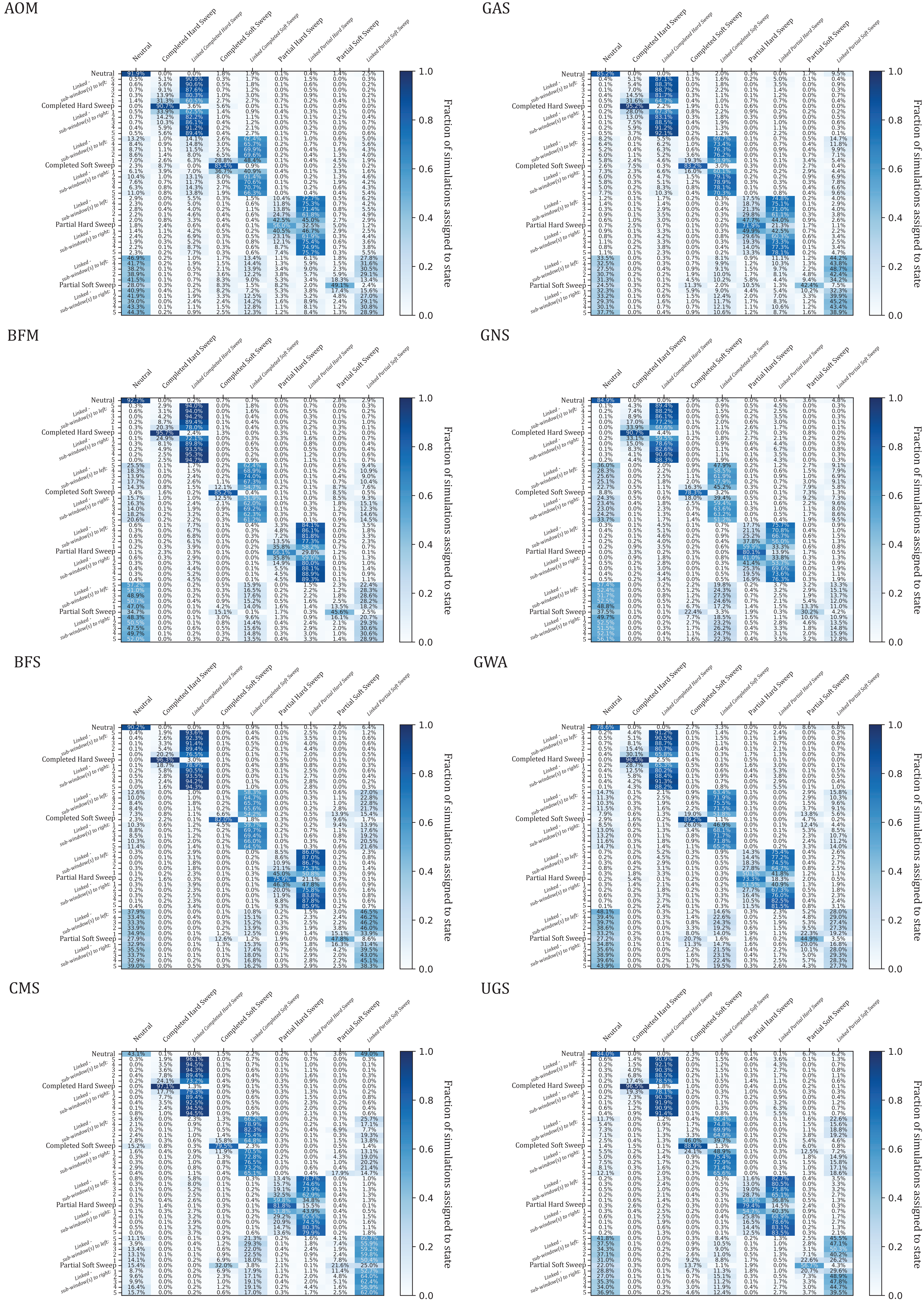
AOM
GAS
BFM
GNS
BFS
GWA
CMS
UGS
Neutral
Completed Hard Sweep
Linked Completed Hard Sweep
Completed Soft Sweep
Linked Completed Soft Sweep
Partial Hard Sweep
Linked Partial Hard Sweep
Partial Soft Sweep
Linked Partial Soft Sweep
Linked - sub-window(s) to left:
Linked - sub-window(s) to right:
Fraction of simulations assigned to state
0.0
0.2
0.4
0.6
0.8
1.0

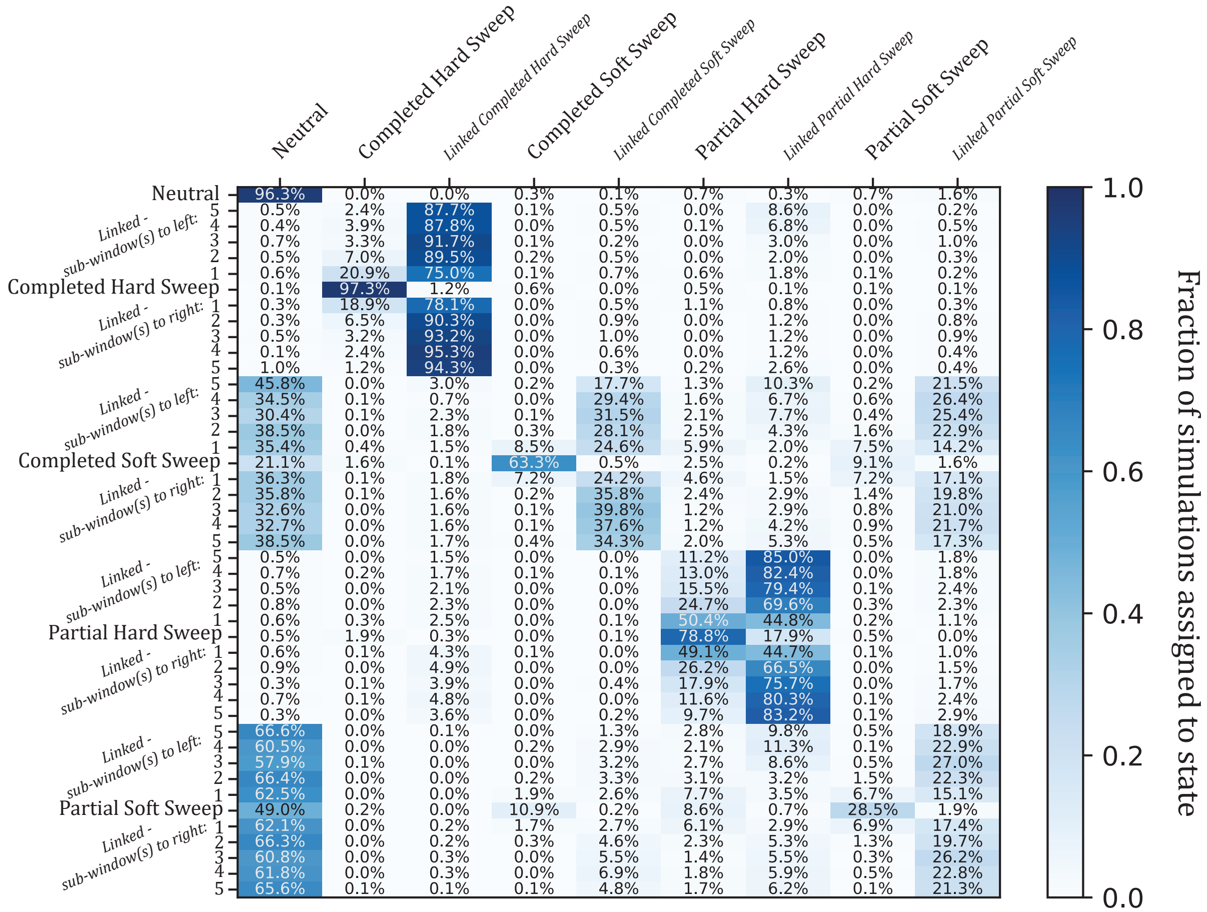

Neutral
Completed Hard Sweep
Linked Completed Hard Sweep
Completed Soft Sweep
Linked Completed Soft Sweep
Partial Hard Sweep
Linked Partial Hard Sweep
Partial Soft Sweep
Linked Partial Soft Sweep
Neutral 96.3% 0.0% 0.0% 0.3% 0.1% 0.7% 0.3% 0.7% 1.6%
Linked - sub-window(s) to left:
5 0.5% 2.4% 87.7% 0.1% 0.5% 0.0% 8.6% 0.0% 0.2%
4 0.4% 3.9% 87.8% 0.0% 0.5% 0.1% 6.8% 0.0% 0.5%
3 0.7% 3.3% 91.7% 0.1% 0.2% 0.0% 3.0% 0.0% 1.0%
2 0.5% 7.0% 89.5% 0.2% 0.5% 0.0% 2.0% 0.0% 0.3%
1 0.6% 20.9% 75.0% 0.1% 0.7% 0.6% 1.8% 0.1% 0.2%
Completed Hard Sweep 0.1% 97.3% 1.2% 0.6% 0.0% 0.5% 0.1% 0.1% 0.1%
Linked - sub-window(s) to right:
1 0.3% 18.9% 78.1% 0.0% 0.5% 1.1% 0.8% 0.0% 0.3%
2 0.3% 6.5% 90.3% 0.0% 0.9% 0.0% 1.2% 0.0% 0.8%
3 0.5% 3.2% 93.2% 0.0% 1.0% 0.0% 1.2% 0.0% 0.9%
4 0.1% 2.4% 95.3% 0.0% 0.6% 0.0% 1.2% 0.0% 0.4%
5 1.0% 1.2% 94.3% 0.0% 0.3% 0.2% 2.6% 0.0% 0.4%
Linked - sub-window(s) to left:
5 45.8% 0.0% 3.0% 0.2% 17.7% 1.3% 10.3% 0.2% 21.5%
4 34.5% 0.1% 0.7% 0.0% 29.4% 1.6% 6.7% 0.6% 26.4%
3 30.4% 0.1% 2.3% 0.1% 31.5% 2.1% 7.7% 0.4% 25.4%
2 38.5% 0.0% 1.8% 0.3% 28.1% 2.5% 4.3% 1.6% 22.9%
1 35.4% 0.4% 1.5% 8.5% 24.6% 5.9% 2.0% 7.5% 14.2%
Completed Soft Sweep 21.1% 1.6% 0.1% 63.3% 0.5% 2.5% 0.2% 9.1% 1.6%
Linked - sub-window(s) to right:
1 36.3% 0.1% 1.8% 7.2% 24.2% 4.6% 1.5% 7.2% 17.1%
2 35.8% 0.1% 1.6% 0.2% 35.8% 2.4% 2.9% 1.4% 19.8%
3 32.6% 0.0% 1.6% 0.1% 39.8% 1.2% 2.9% 0.8% 21.0%
4 32.7% 0.0% 1.6% 0.1% 37.6% 1.2% 4.2% 0.9% 21.7%
5 38.5% 0.0% 1.7% 0.4% 34.3% 2.0% 5.3% 0.5% 17.3%
Linked - sub-window(s) to left:
5 0.5% 0.0% 1.5% 0.0% 0.0% 11.2% 85.0% 0.0% 1.8%
4 0.7% 0.2% 1.7% 0.1% 0.1% 13.0% 82.4% 0.0% 1.8%
3 0.5% 0.0% 2.1% 0.0% 0.0% 15.5% 79.4% 0.1% 2.4%
2 0.8% 0.0% 2.3% 0.0% 0.0% 24.7% 69.6% 0.3% 2.3%
1 0.6% 0.3% 2.5% 0.0% 0.1% 50.4% 44.8% 0.2% 1.1%
Partial Hard Sweep 0.5% 1.9% 0.3% 0.0% 0.1% 78.8% 17.9% 0.5% 0.0%
Linked - sub-window(s) to right:
1 0.6% 0.1% 4.3% 0.1% 0.0% 49.1% 44.7% 0.1% 1.0%
2 0.9% 0.0% 4.9% 0.0% 0.0% 26.2% 66.5% 0.0% 1.5%
3 0.3% 0.1% 3.9% 0.0% 0.4% 17.9% 75.7% 0.0% 1.7%
4 0.7% 0.1% 4.8% 0.0% 0.0% 11.6% 80.3% 0.1% 2.4%
5 0.3% 0.0% 3.6% 0.0% 0.2% 9.7% 83.2% 0.1% 2.9%
Linked - sub-window(s) to left:
5 66.6% 0.0% 0.1% 0.0% 1.3% 2.8% 9.8% 0.5% 18.9%
4 60.5% 0.0% 0.0% 0.2% 2.9% 2.1% 11.3% 0.1% 22.9%
3 57.9% 0.1% 0.0% 0.0% 3.2% 2.7% 8.6% 0.5% 27.0%
2 66.4% 0.0% 0.0% 0.2% 3.3% 3.1% 3.2% 1.5% 22.3%
1 62.5% 0.0% 0.0% 1.9% 2.6% 7.7% 3.5% 6.7% 15.1%
Partial Soft Sweep 49.0% 0.2% 0.0% 10.9% 0.2% 8.6% 0.7% 28.5% 1.9%
Linked - sub-window(s) to right:
1 62.1% 0.0% 0.2% 1.7% 2.7% 6.1% 2.9% 6.9% 17.4%
2 66.3% 0.0% 0.2% 0.3% 4.6% 2.3% 5.3% 1.3% 19.7%
3 60.8% 0.0% 0.3% 0.0% 5.5% 1.4% 5.5% 0.3% 26.2%
4 61.8% 0.0% 0.3% 0.0% 6.9% 1.8% 5.9% 0.5% 22.8%
5 65.6% 0.1% 0.1% 0.1% 4.8% 1.7% 6.2% 0.1% 21.3%
1.0
0.8
0.6
0.4
0.2
0.0
Fraction of simulations assigned to state

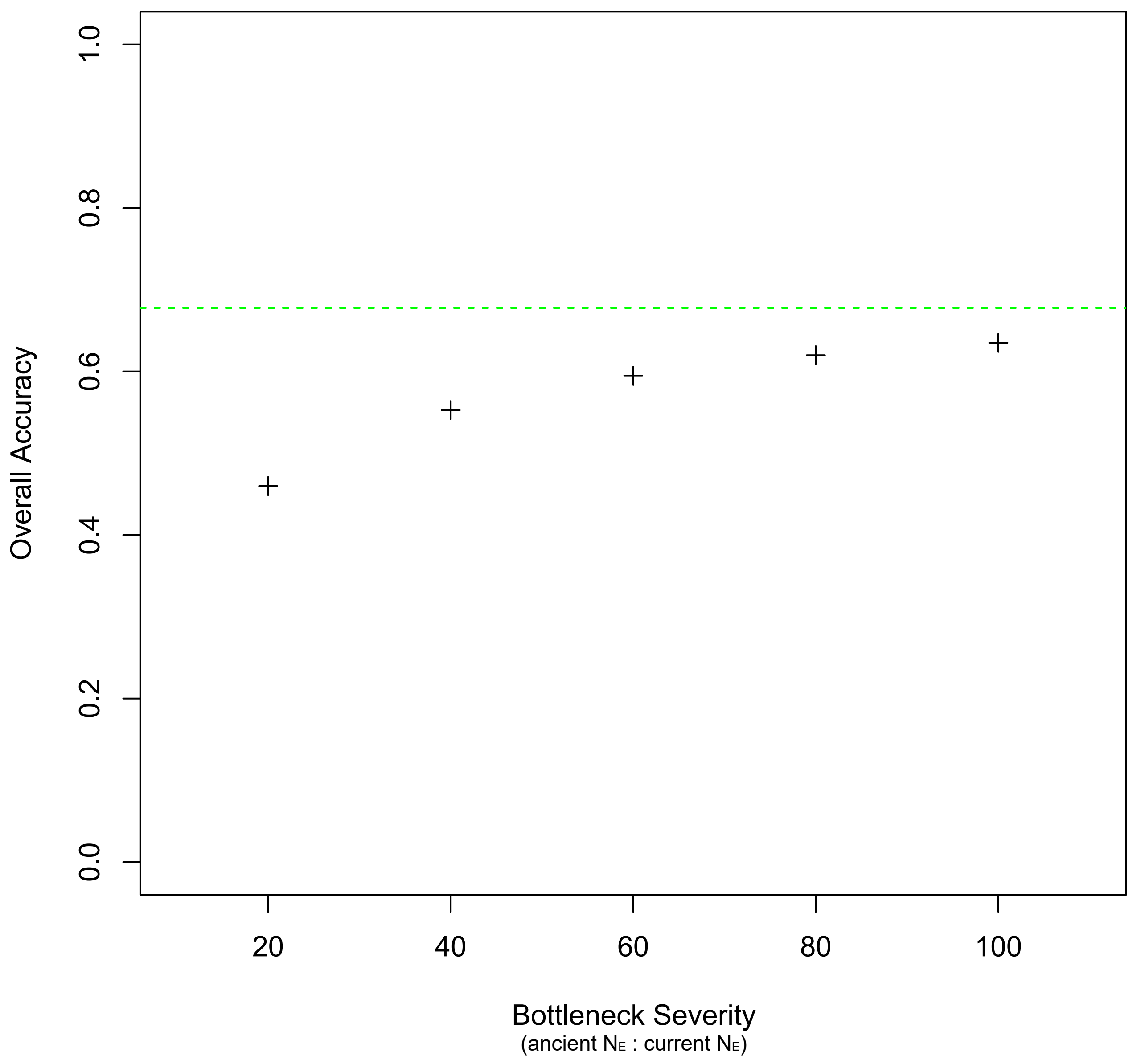

Overall Accuracy
0.0
0.2
0.4
0.6
0.8
1.0
20
40
60
80
100
Bottleneck Severity
(ancient NE : current NE)

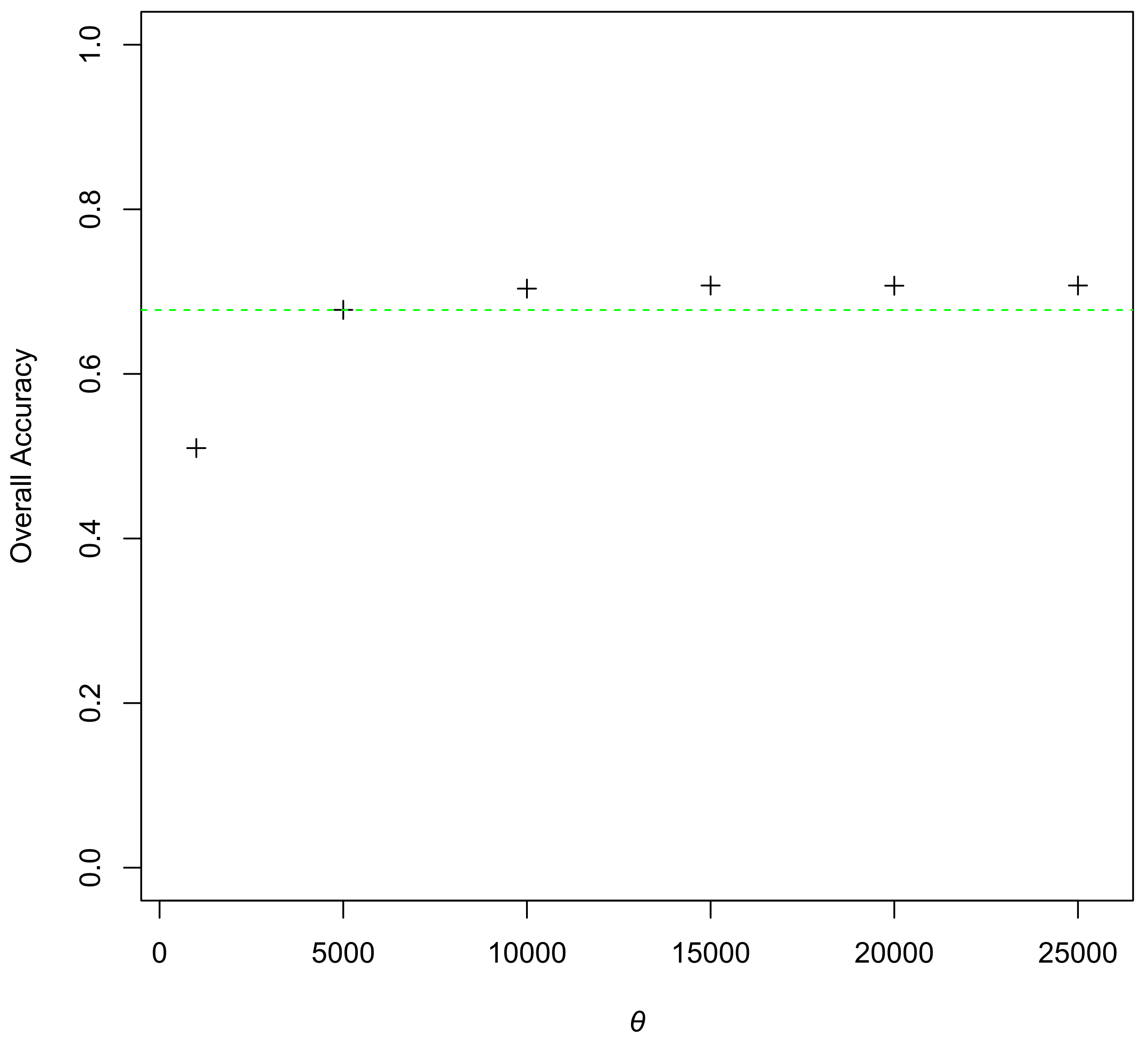

Overall Accuracy
0.0
0.2
0.4
0.6
0.8
1.0
0
5000
10000
15000
20000
25000
θ

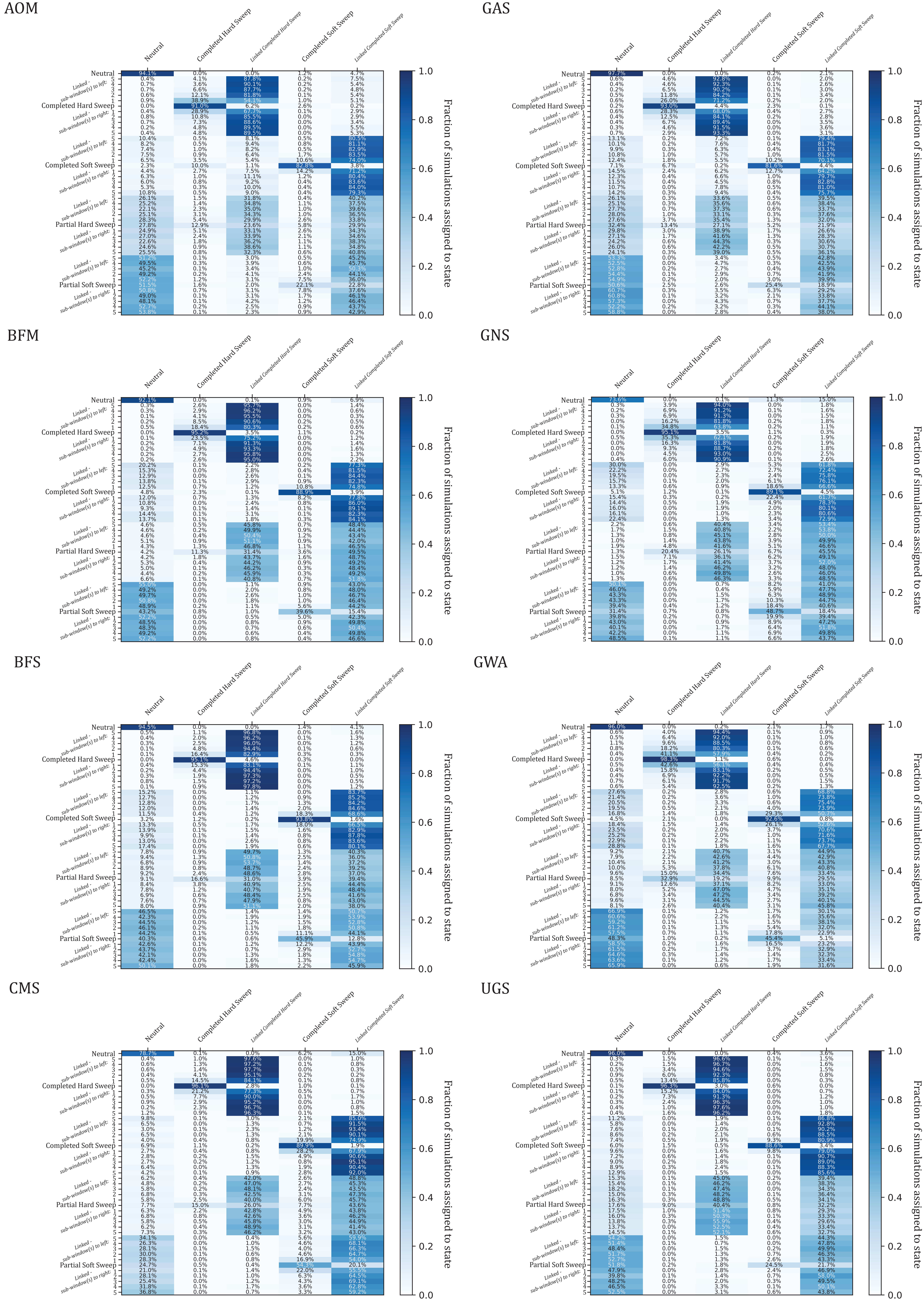

AOM
GAS
BFM
GNS
BFS
GWA
CMS
UGS
Neutral
Completed Hard Sweep
Linked Completed Hard Sweep
Completed Soft Sweep
Linked Completed Soft Sweep
Linked - sub-window(s) to left:
Linked - sub-window(s) to right:
Partial Hard Sweep
Partial Soft Sweep
Fraction of simulations assigned to state
0.0
0.2
0.4
0.6
0.8
1.0

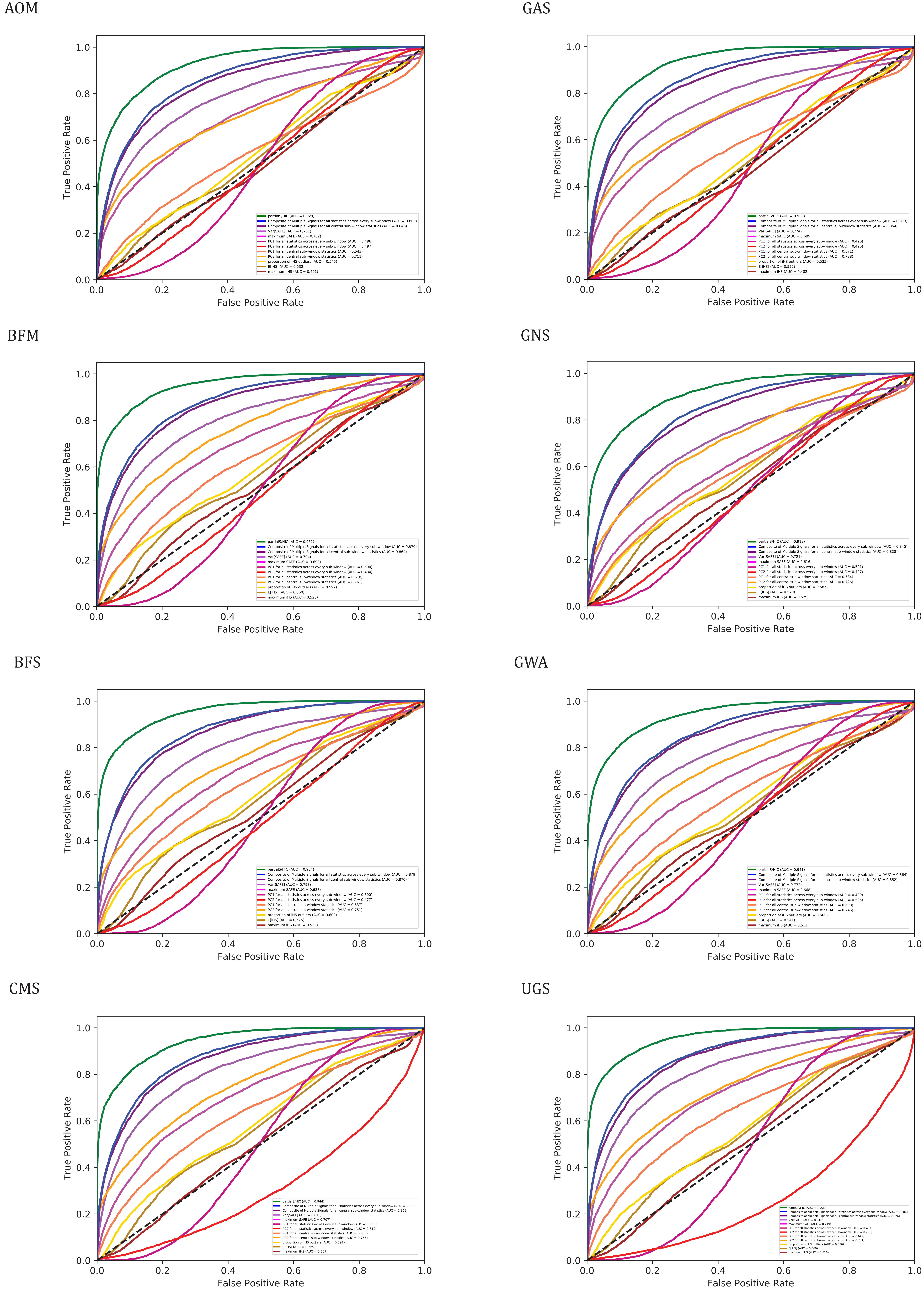

AOM
True Positive Rate
False Positive Rate
partialS/HIC (AUC = 0.929)
Composite of Multiple Signals for all statistics across every sub-window (AUC = 0.863)
Composite of Multiple Signals for all central sub-window statistics (AUC = 0.846)
Var[SAFE] (AUC = 0.781)
maximum SAFE (AUC = 0.702)
PC1 for all statistics across every sub-window (AUC = 0.498)
PC2 for all statistics across every sub-window (AUC = 0.497)
PC1 for all central sub-window statistics (AUC = 0.543)
PC2 for all central sub-window statistics (AUC = 0.711)
proportion of iHS outliers (AUC = 0.545)
E[iHS] (AUC = 0.532)
maximum iHS (AUC = 0.491)
GAS
True Positive Rate
False Positive Rate
partialS/HIC (AUC = 0.938)
Composite of Multiple Signals for all statistics across every sub-window (AUC = 0.873)
Composite of Multiple Signals for all central sub-window statistics (AUC = 0.854)
Var[SAFE] (AUC = 0.774)
maximum SAFE (AUC = 0.699)
PC1 for all statistics across every sub-window (AUC = 0.496)
PC2 for all statistics across every sub-window (AUC = 0.496)
PC1 for all central sub-window statistics (AUC = 0.571)
PC2 for all central sub-window statistics (AUC = 0.728)
proportion of iHS outliers (AUC = 0.535)
E[iHS] (AUC = 0.522)
maximum iHS (AUC = 0.482)
BFM
True Positive Rate
False Positive Rate
partialS/HIC (AUC = 0.952)
Composite of Multiple Signals for all statistics across every sub-window (AUC = 0.879)
Composite of Multiple Signals for all central sub-window statistics (AUC = 0.864)
Var[SAFE] (AUC = 0.794)
maximum SAFE (AUC = 0.692)
PC1 for all statistics across every sub-window (AUC = 0.500)
PC2 for all statistics across every sub-window (AUC = 0.484)
PC1 for all central sub-window statistics (AUC = 0.618)
PC2 for all central sub-window statistics (AUC = 0.761)
proportion of iHS outliers (AUC = 0.592)
E[iHS] (AUC = 0.560)
maximum iHS (AUC = 0.520)
GNS
True Positive Rate
False Positive Rate
partialS/HIC (AUC = 0.918)
Composite of Multiple Signals for all statistics across every sub-window (AUC = 0.845)
Composite of Multiple Signals for all central sub-window statistics (AUC = 0.828)
Var[SAFE] (AUC = 0.721)
maximum SAFE (AUC = 0.616)
PC1 for all statistics across every sub-window (AUC = 0.501)
PC2 for all statistics across every sub-window (AUC = 0.497)
PC1 for all central sub-window statistics (AUC = 0.584)
PC2 for all central sub-window statistics (AUC = 0.726)
proportion of iHS outliers (AUC = 0.587)
E[iHS] (AUC = 0.570)
maximum iHS (AUC = 0.529)
BFS
True Positive Rate
False Positive Rate
partialS/HIC (AUC = 0.954)
Composite of Multiple Signals for all statistics across every sub-window (AUC = 0.879)
Composite of Multiple Signals for all central sub-window statistics (AUC = 0.870)
Var[SAFE] (AUC = 0.793)
maximum SAFE (AUC = 0.687)
PC1 for all statistics across every sub-window (AUC = 0.500)
PC2 for all statistics across every sub-window (AUC = 0.477)
PC1 for all central sub-window statistics (AUC = 0.637)
PC2 for all central sub-window statistics (AUC = 0.751)
proportion of iHS outliers (AUC = 0.602)
E[iHS] (AUC = 0.575)
maximum iHS (AUC = 0.533)
GWA
True Positive Rate
False Positive Rate
partialS/HIC (AUC = 0.941)
Composite of Multiple Signals for all statistics across every sub-window (AUC = 0.864)
Composite of Multiple Signals for all central sub-window statistics (AUC = 0.852)
Var[SAFE] (AUC = 0.772)
maximum SAFE (AUC = 0.668)
PC1 for all statistics across every sub-window (AUC = 0.499)
PC2 for all statistics across every sub-window (AUC = 0.505)
PC1 for all central sub-window statistics (AUC = 0.598)
PC2 for all central sub-window statistics (AUC = 0.746)
proportion of iHS outliers (AUC = 0.565)
E[iHS] (AUC = 0.541)
maximum iHS (AUC = 0.512)
CMS
True Positive Rate
False Positive Rate
partialS/HIC (AUC = 0.944)
Composite of Multiple Signals for all statistics across every sub-window (AUC = 0.880)
Composite of Multiple Signals for all central sub-window statistics (AUC = 0.869)
Var[SAFE] (AUC = 0.813)
maximum SAFE (AUC = 0.707)
PC1 for all statistics across every sub-window (AUC = 0.505)
PC2 for all statistics across every sub-window (AUC = 0.319)
PC1 for all central sub-window statistics (AUC = 0.635)
PC2 for all central sub-window statistics (AUC = 0.755)
proportion of iHS outliers (AUC = 0.591)
E[iHS] (AUC = 0.569)
maximum iHS (AUC = 0.507)
UGS
True Positive Rate
False Positive Rate
partialS/HIC (AUC = 0.958)
Composite of Multiple Signals for all statistics across every sub-window (AUC = 0.886)
Composite of Multiple Signals for all central sub-window statistics (AUC = 0.876)
Var[SAFE] (AUC = 0.818)
maximum SAFE (AUC = 0.719)
PC1 for all statistics across every sub-window (AUC = 0.497)
PC2 for all statistics across every sub-window (AUC = 0.268)
PC1 for all central sub-window statistics (AUC = 0.642)
PC2 for all central sub-window statistics (AUC = 0.751)
proportion of iHS outliers (AUC = 0.576)
E[iHS] (AUC = 0.560)
maximum iHS (AUC = 0.516)

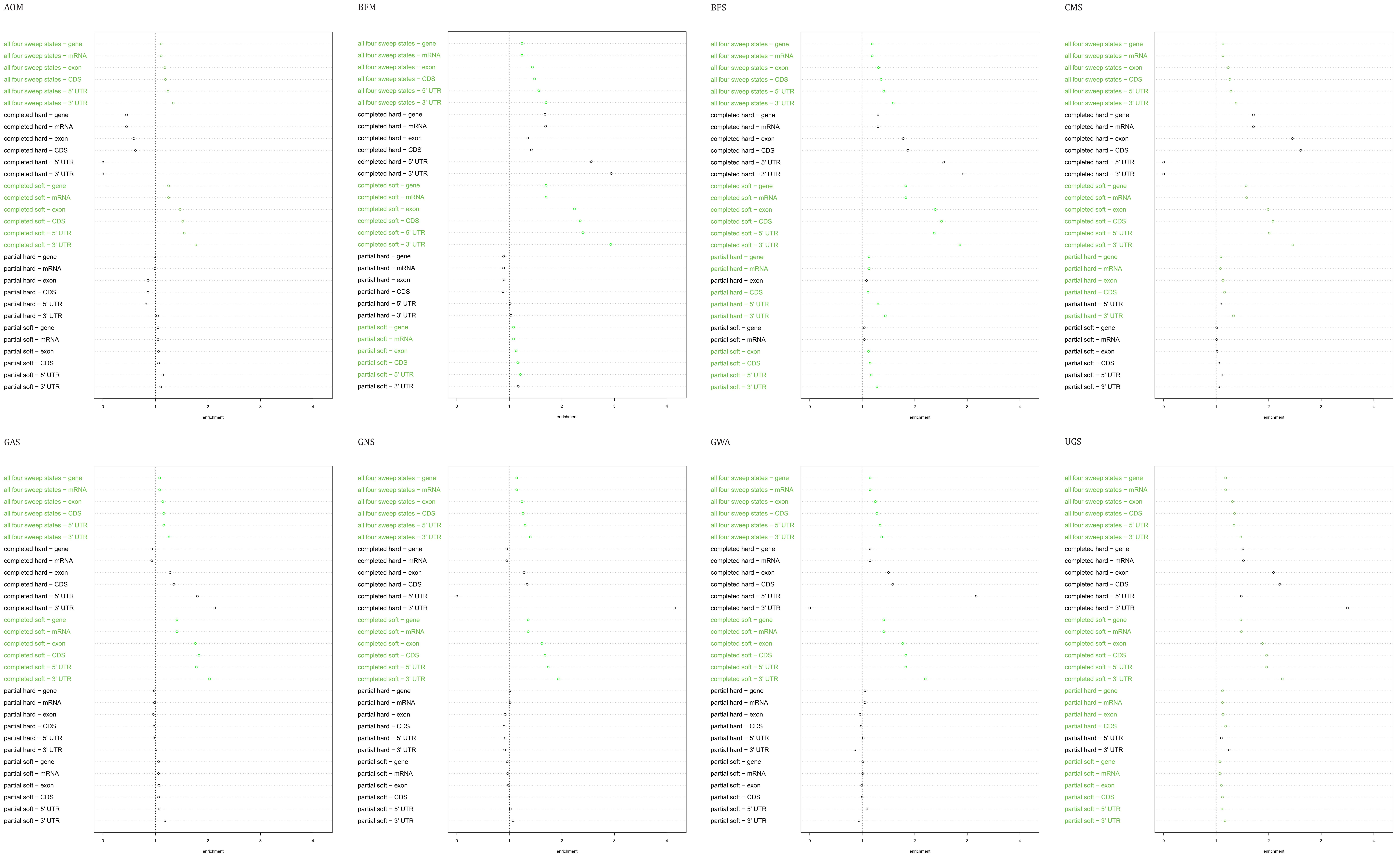
AOM
BFM
BFS
CMS
GAS
GNS
GWA
UGS
all four sweep states – gene
all four sweep states – mRNA
all four sweep states – exon
all four sweep states – CDS
all four sweep states – 5' UTR
all four sweep states – 3' UTR
completed hard – gene
completed hard – mRNA
completed hard – exon
completed hard – CDS
completed hard – 5' UTR
completed hard – 3' UTR
completed soft – gene
completed soft – mRNA
completed soft – exon
completed soft – CDS
completed soft – 5' UTR
completed soft – 3' UTR
partial hard – gene
partial hard – mRNA
partial hard – exon
partial hard – CDS
partial hard – 5' UTR
partial hard – 3' UTR
partial soft – gene
partial soft – mRNA
partial soft – exon
partial soft – CDS
partial soft – 5' UTR
partial soft – 3' UTR
0
1
2
3
4
enrichment

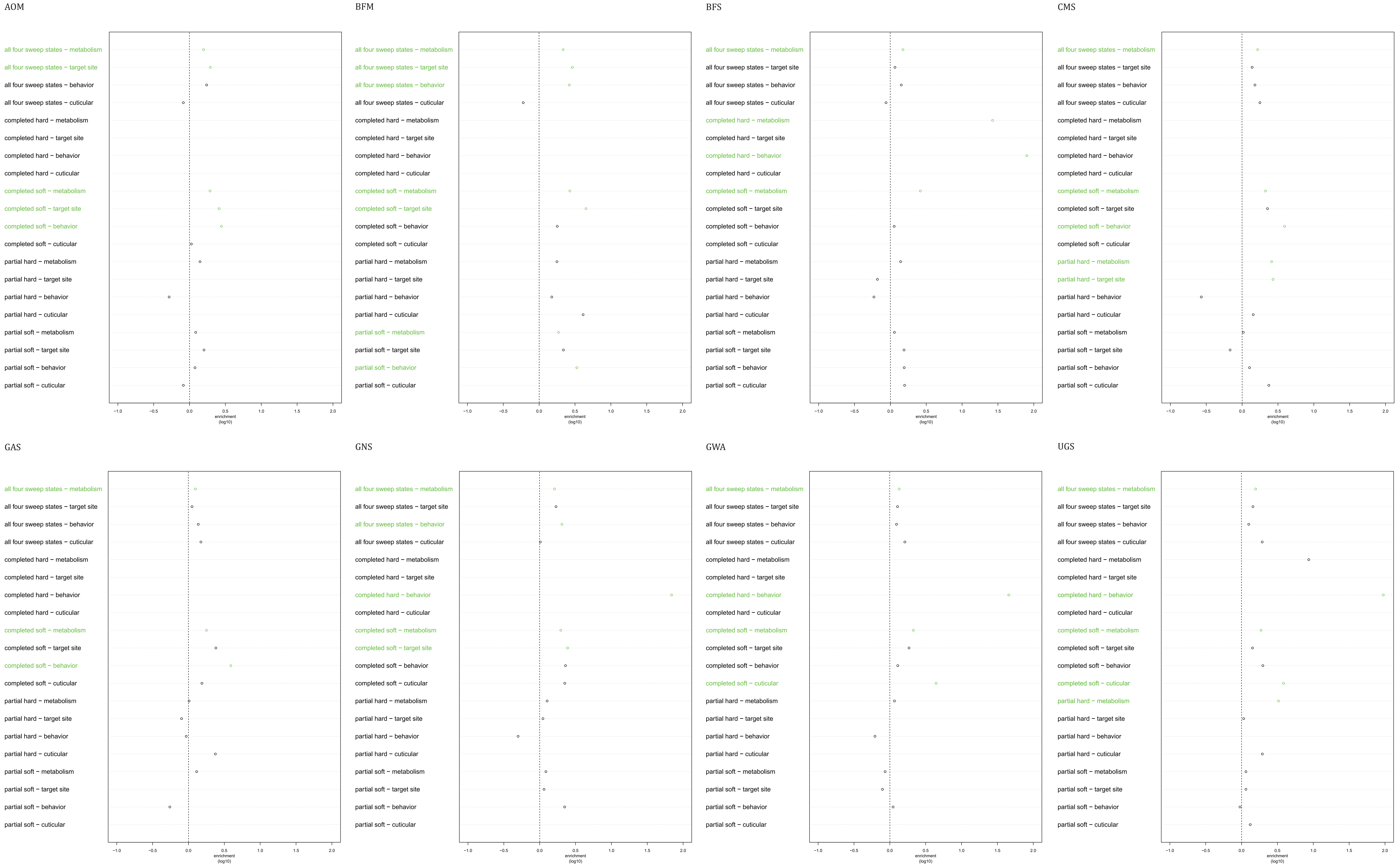
AOM
all four sweep states − metabolism
all four sweep states − target site
all four sweep states − behavior
all four sweep states − cuticular
completed hard − metabolism
completed hard − target site
completed hard − behavior
completed hard − cuticular
completed soft − metabolism
completed soft − target site
completed soft − behavior
completed soft − cuticular
partial hard − metabolism
partial hard − target site
partial hard − behavior
partial hard − cuticular
partial soft − metabolism
partial soft − target site
partial soft − behavior
partial soft − cuticular
−1.0 −0.5 0.0 0.5 1.0 1.5 2.0
enrichment
(log10)
BFM
all four sweep states − metabolism
all four sweep states − target site
all four sweep states − behavior
all four sweep states − cuticular
completed hard − metabolism
completed hard − target site
completed hard − behavior
completed hard − cuticular
completed soft − metabolism
completed soft − target site
completed soft − behavior
completed soft − cuticular
partial hard − metabolism
partial hard − target site
partial hard − behavior
partial hard − cuticular
partial soft − metabolism
partial soft − target site
partial soft − behavior
partial soft − cuticular
−1.0 −0.5 0.0 0.5 1.0 1.5 2.0
enrichment
(log10)
BFS
all four sweep states − metabolism
all four sweep states − target site
all four sweep states − behavior
all four sweep states − cuticular
completed hard − metabolism
completed hard − target site
completed hard − behavior
completed hard − cuticular
completed soft − metabolism
completed soft − target site
completed soft − behavior
completed soft − cuticular
partial hard − metabolism
partial hard − target site
partial hard − behavior
partial hard − cuticular
partial soft − metabolism
partial soft − target site
partial soft − behavior
partial soft − cuticular
−1.0 −0.5 0.0 0.5 1.0 1.5 2.0
enrichment
(log10)
CMS
all four sweep states − metabolism
all four sweep states − target site
all four sweep states − behavior
all four sweep states − cuticular
completed hard − metabolism
completed hard − target site
completed hard − behavior
completed hard − cuticular
completed soft − metabolism
completed soft − target site
completed soft − behavior
completed soft − cuticular
partial hard − metabolism
partial hard − target site
partial hard − behavior
partial hard − cuticular
partial soft − metabolism
partial soft − target site
partial soft − behavior
partial soft − cuticular
−1.0 −0.5 0.0 0.5 1.0 1.5 2.0
enrichment
(log10)
GAS
all four sweep states − metabolism
all four sweep states − target site
all four sweep states − behavior
all four sweep states − cuticular
completed hard − metabolism
completed hard − target site
completed hard − behavior
completed hard − cuticular
completed soft − metabolism
completed soft − target site
completed soft − behavior
completed soft − cuticular
partial hard − metabolism
partial hard − target site
partial hard − behavior
partial hard − cuticular
partial soft − metabolism
partial soft − target site
partial soft − behavior
partial soft − cuticular
−1.0 −0.5 0.0 0.5 1.0 1.5 2.0
enrichment
(log10)
GNS
all four sweep states − metabolism
all four sweep states − target site
all four sweep states − behavior
all four sweep states − cuticular
completed hard − metabolism
completed hard − target site
completed hard − behavior
completed hard − cuticular
completed soft − metabolism
completed soft − target site
completed soft − behavior
completed soft − cuticular
partial hard − metabolism
partial hard − target site
partial hard − behavior
partial hard − cuticular
partial soft − metabolism
partial soft − target site
partial soft − behavior
partial soft − cuticular
−1.0 −0.5 0.0 0.5 1.0 1.5 2.0
enrichment
(log10)
GWA
all four sweep states − metabolism
all four sweep states − target site
all four sweep states − behavior
all four sweep states − cuticular
completed hard − metabolism
completed hard − target site
completed hard − behavior
completed hard − cuticular
completed soft − metabolism
completed soft − target site
completed soft − behavior
completed soft − cuticular
partial hard − metabolism
partial hard − target site
partial hard − behavior
partial hard − cuticular
partial soft − metabolism
partial soft − target site
partial soft − behavior
partial soft − cuticular
−1.0 −0.5 0.0 0.5 1.0 1.5 2.0
enrichment
(log10)
UGS
all four sweep states − metabolism
all four sweep states − target site
all four sweep states − behavior
all four sweep states − cuticular
completed hard − metabolism
completed hard − target site
completed hard − behavior
completed hard − cuticular
completed soft − metabolism
completed soft − target site
completed soft − behavior
completed soft − cuticular
partial hard − metabolism
partial hard − target site
partial hard − behavior
partial hard − cuticular
partial soft − metabolism
partial soft − target site
partial soft − behavior
partial soft − cuticular
−1.0 −0.5 0.0 0.5 1.0 1.5 2.0
enrichment
(log10)
