# Supplementary material for: Discovery of Ongoing Selective Sweeps within Anopheles Mosquito Populations Using Deep Learning
Source: Mol Biol Evol. 2020 Oct 6;38(3):1168–83. doi: 10.1093/molbev/msaa259 (PMC7947845; doi:10.1093/molbev/msaa259)
Supplement: msaa259_Supplementary_Data [file msaa259_supplementary_data.zip › Tables S1-5.pdf]

Table S1. Accuracy rate for deep learning *in silico* test per simulated population dataset.

Population	Accuracy
AOM	0.610
BFM	0.661
BFS	0.691
CMS	0.711
GAS	0.667
GNS	0.546
GWA	0.625
UGS	0.678
median	0.664
mean	0.648

Table S2. Accuracy rate for five-state deep learning *in silico* test per simulated population dataset (neutral regions and completed sweeps only).

**Population**	**Accuracy**
AOM	0.822
BFM	0.872
BFS	0.881
CMS	0.897
GAS	0.830
GNS	0.792
GWA	0.780
UGS	0.906
median	0.851
mean	0.848

Table S3. Nine-state calls for individual chromosome arms and genome-wide for each empirical mosquito population dataset.

	AOM	BFM	BFS	CMS	GAS	GNS	GWA	UGS	*median*	*mean*
	neutral *(proportion)*									
**2L**	0.4524	0.6649	0.2929	0.0167	0.3153	0.2960	0.0395	0.1990	0.2944	0.2846
**2R**	0.4217	0.3822	0.2899	0.0046	0.2227	0.3465	0.0335	0.2231	0.2565	0.2405
**3L**	0.5452	0.6728	0.4012	0.0067	0.2709	0.5540	0.3315	0.3663	0.3838	0.3936
**3R**	0.5181	0.6202	0.3123	0.0069	0.2676	0.4124	0.2433	0.3327	0.3225	0.3392
**genome-wide**	0.4767	0.5608	0.3172	0.0081	0.2626	0.3912	0.1483	0.2761	0.2967	0.3051
	completed hard *(proportion)*									
**2L**	0.0000	0.0000	0.0000	0.0006	0.0004	0.0000	0.0000	0.0004	0.0000	0.0002
**2R**	0.0010	0.0004	0.0002	0.0000	0.0002	0.0002	0.0009	0.0002	0.0002	0.0004
**3L**	0.0000	0.0004	0.0000	0.0000	0.0000	0.0000	0.0008	0.0000	0.0000	0.0002
**3R**	0.0000	0.0008	0.0005	0.0000	0.0005	0.0003	0.0003	0.0007	0.0004	0.0004
**genome-wide**	0.0003	0.0004	0.0002	0.0001	0.0003	0.0002	0.0005	0.0004	0.0003	0.0003
	completed hard – linked *(proportion)*									
**2L**	0.0004	0.0004	0.0017	0.0035	0.0000	0.0019	0.0000	0.0007	0.0006	0.0011
**2R**	0.0045	0.0018	0.0013	0.0034	0.0016	0.0009	0.0018	0.0024	0.0018	0.0022
**3L**	0.0010	0.0020	0.0004	0.0027	0.0005	0.0013	0.0004	0.0008	0.0009	0.0011
**3R**	0.0028	0.0025	0.0020	0.0007	0.0003	0.0000	0.0000	0.0012	0.0009	0.0012
**genome-wide**	0.0026	0.0017	0.0014	0.0025	0.0007	0.0009	0.0007	0.0014	0.0014	0.0015
	completed soft *(proportion)*									
**2L**	0.0587	0.0309	0.0187	0.0273	0.0393	0.0596	0.0665	0.0540	0.0466	0.0444
**2R**	0.0763	0.0307	0.0254	0.0259	0.0664	0.0580	0.0817	0.0647	0.0614	0.0536
**3L**	0.0558	0.0242	0.0234	0.0403	0.0332	0.0511	0.0676	0.0547	0.0457	0.0438
**3R**	0.0446	0.0257	0.0168	0.0329	0.0304	0.0520	0.0716	0.0522	0.0388	0.0408
**genome-wide**	0.0601	0.0281	0.0212	0.0310	0.0449	0.0554	0.0732	0.0571	0.0501	0.0464
	completed soft – linked *(proportion)*									
**2L**	0.1106	0.0872	0.1333	0.2428	0.1537	0.3515	0.3744	0.2050	0.1793	0.2073
**2R**	0.1267	0.1635	0.1310	0.2699	0.2286	0.2983	0.4573	0.1666	0.1976	0.2302
**3L**	0.0837	0.0890	0.1257	0.3544	0.1034	0.2003	0.2353	0.1480	0.1368	0.1675
**3R**	0.0823	0.0920	0.1310	0.3211	0.0783	0.2407	0.2631	0.1572	0.1441	0.1707
**genome-wide**	0.1033	0.1139	0.1305	0.2955	0.1487	0.2755	0.3448	0.1682	0.1584	0.1975
	partial hard *(proportion)*									
**2L**	0.0281	0.0103	0.0463	0.0331	0.1380	0.0331	0.0395	0.0427	0.0363	0.0464
**2R**	0.0363	0.0241	0.0554	0.0836	0.0917	0.0359	0.0276	0.0293	0.0361	0.0480
**3L**	0.0168	0.0071	0.0138	0.0113	0.1624	0.0074	0.0118	0.0119	0.0118	0.0303
**3R**	0.0237	0.0135	0.0388	0.0176	0.1676	0.0315	0.0193	0.0113	0.0215	0.0404
**genome-wide**	0.0277	0.0151	0.0412	0.0405	0.1350	0.0291	0.0248	0.0236	0.0284	0.0422
	partial hard – linked *(proportion)*									
**2L**	0.0768	0.0223	0.1382	0.0864	0.1325	0.0581	0.0512	0.0823	0.0796	0.0810

**2R**	0.1011	0.0767	0.1338	0.1535	0.1334	0.0882	0.0372	0.0533	0.0947	0.0971
**3L**	0.0635	0.0115	0.0461	0.0329	0.1867	0.0234	0.0248	0.0184	0.0289	0.0509
**3R**	0.0811	0.0353	0.1112	0.0652	0.1821	0.0746	0.0341	0.0204	0.0699	0.0755
**genome-wide**	0.0841	0.0419	0.1122	0.0914	0.1564	0.0670	0.0370	0.0433	0.0755	0.0791
	partial soft *(proportion)*									
**2L**	0.0885	0.0475	0.0598	0.1040	0.0531	0.0510	0.1170	0.1146	0.0741	0.0794
**2R**	0.0708	0.0504	0.0737	0.0535	0.0499	0.0426	0.1024	0.1292	0.0621	0.0716
**3L**	0.0659	0.0461	0.0734	0.0839	0.0538	0.0438	0.1105	0.1296	0.0696	0.0759
**3R**	0.0761	0.0473	0.0702	0.1547	0.0545	0.0426	0.1249	0.1447	0.0732	0.0894
**genome-wide**	0.0751	0.0481	0.0698	0.0986	0.0526	0.0445	0.1133	0.1308	0.0724	0.0791
	partial soft – linked *(proportion)*									
**2L**	0.1846	0.1365	0.3092	0.4855	0.1678	0.1489	0.3119	0.3013	0.2429	0.2557
**2R**	0.1616	0.2704	0.2893	0.4055	0.2054	0.1293	0.2575	0.3312	0.2640	0.2563
**3L**	0.1683	0.1469	0.3159	0.4679	0.1891	0.1188	0.2172	0.2704	0.2031	0.2368
**3R**	0.1712	0.1629	0.3173	0.4010	0.2186	0.1459	0.2436	0.2795	0.2311	0.2425
**genome-wide**	0.1702	0.1898	0.3062	0.4322	0.1989	0.1362	0.2575	0.2991	0.2282	0.2487
	neutral *(count)*									
**2L**	1,125	1,875	848	52	802	795	108	568	798.5	771.6
**2R**	1,764	1,730	1,361	23	950	1,552	147	1,034	1,197.5	1,070.1
**3L**	1,134	1,694	1,044	20	579	1,278	789	958	1,001.0	937.0
**3R**	1,858	2,441	1,264	31	977	1,569	935	1,352	1,308.0	1,303.4
**genome-wide**	5,881	7,740	4,517	126	3,308	5,194	1,979	3,912	4,214.5	4,082.1
	completed hard *(count)*									
**2L**	0	0	0	2	1	0	0	1	0.0	0.5
**2R**	4	2	1	0	1	1	4	1	1.0	1.8
**3L**	0	1	0	0	0	0	2	0	0.0	0.4
**3R**	0	3	2	0	2	1	1	3	1.5	1.5
**genome-wide**	4	6	3	2	4	2	7	5	4.0	4.1
	completed hard – linked *(count)*									
**2L**	1	1	5	11	0	5	0	2	1.5	3.1
**2R**	19	8	6	17	7	4	8	11	8.0	10.0
**3L**	2	5	1	8	1	3	1	2	2.0	2.9
**3R**	10	10	8	3	1	0	0	5	4.0	4.6
**genome-wide**	32	24	20	39	9	12	9	20	20.0	20.6
	completed soft *(count)*									
**2L**	146	87	54	85	100	160	182	154	123.0	121.0
**2R**	319	139	119	129	283	260	358	300	271.5	238.4
**3L**	116	61	61	121	71	118	161	143	117.0	106.5
**3R**	160	101	68	148	111	198	275	212	154.0	159.1
**genome-wide**	741	388	302	483	565	736	976	809	650.5	625.0
	completed soft – linked *(count)*									
**2L**	275	246	386	756	391	944	1,024	585	488.0	575.9

**2R**	530	740	615	1,343	975	1,336	2,005	772	873.5	1,039.5
**3L**	174	224	327	1,065	221	462	560	387	357.0	427.5
**3R**	295	362	530	1,443	286	916	1,011	639	584.5	685.3
**genome-wide**	1,274	1,572	1,858	4,607	1,873	3,658	4,600	2,383	2,128.0	2,728.1
	partial hard *(count)*									
**2L**	70	29	134	103	351	89	108	122	105.5	125.8
**2R**	152	109	260	416	391	161	121	136	156.5	218.3
**3L**	35	18	36	34	347	17	28	31	32.5	68.3
**3R**	85	53	157	79	612	120	74	46	82.0	153.3
**genome-wide**	342	209	587	632	1,701	387	331	335	364.5	565.5
	partial hard – linked *(count)*									
**2L**	191	63	400	269	337	156	140	235	213.0	223.9
**2R**	423	347	628	764	569	395	163	247	409.0	442.0
**3L**	132	29	120	99	399	54	59	48	79.0	117.5
**3R**	291	139	450	293	665	284	131	83	287.5	292.0
**genome-wide**	1,037	578	1,598	1,425	1,970	889	493	613	963.0	1,075.4
	partial soft *(count)*									
**2L**	220	134	173	324	135	137	320	327	196.5	221.3
**2R**	296	228	346	266	213	191	449	599	281.0	323.5
**3L**	137	116	191	252	115	101	263	339	164.0	189.3
**3R**	273	186	284	695	199	162	480	588	278.5	358.4
**genome-wide**	926	664	994	1,537	662	591	1,512	1,853	960.0	1,092.4
	partial soft – linked *(count)*									
**2L**	459	385	895	1,512	427	400	853	860	656.0	723.9
**2R**	676	1,224	1,358	2,018	876	579	1,129	1,535	1,176.5	1,174.4
**3L**	350	370	822	1,406	404	274	517	707	460.5	606.3
**3R**	614	641	1,284	1,802	798	555	936	1,136	867.0	970.8
**genome-wide**	2,099	2,620	4,359	6,738	2,505	1,808	3,435	4,238	3,027.5	3,475.3
	neutral *(proportion)* *corrected for false discovery*									
**2L**	0.4922	0.7173	0.3247	0.0387	0.3700	0.3486	0.0502	0.2369	0.3367	0.3223
**2R**	0.4589	0.4122	0.3214	0.0107	0.2614	0.4081	0.0427	0.2656	0.2935	0.2726
**3L**	0.5932	0.7257	0.4448	0.0154	0.3180	0.6525	0.4218	0.4361	0.4405	0.4510
**3R**	0.5638	0.6690	0.3463	0.0160	0.3141	0.4857	0.3095	0.3960	0.3712	0.3876
**genome-wide**	0.5188	0.6050	0.3517	0.0188	0.3082	0.4608	0.1887	0.3287	0.3402	0.3476
	completed hard *(proportion)* *corrected for false discovery*									
**2L**	0.0000	0.0000	0.0000	0.0006	0.0004	0.0000	0.0000	0.0004	0.0000	0.0002
**2R**	0.0010	0.0004	0.0002	0.0000	0.0002	0.0002	0.0009	0.0002	0.0002	0.0004
**3L**	0.0000	0.0004	0.0000	0.0000	0.0000	0.0000	0.0008	0.0000	0.0000	0.0002
**3R**	0.0000	0.0008	0.0005	0.0000	0.0005	0.0003	0.0003	0.0007	0.0004	0.0004
**genome-wide**	0.0003	0.0004	0.0002	0.0001	0.0003	0.0002	0.0005	0.0004	0.0003	0.0003

	completed hard – linked *(proportion)* *corrected for false discovery*									
**2L**	0.0004	-0.0011	0.0017	0.0035	0.0000	0.0019	0.0000	0.0007	0.0006	0.0009
**2R**	0.0045	0.0009	0.0013	0.0034	0.0016	0.0009	0.0018	0.0024	0.0017	0.0021
**3L**	0.0010	0.0005	0.0004	0.0027	0.0005	0.0013	0.0004	0.0008	0.0006	0.0009
**3R**	0.0028	0.0012	0.0020	0.0007	0.0003	0.0000	0.0000	0.0012	0.0009	0.0010
**genome-wide**	0.0026	0.0005	0.0014	0.0025	0.0007	0.0009	0.0007	0.0014	0.0012	0.0013
	completed soft *(proportion)* *corrected for false discovery*									
**2L**	0.0498	0.0258	0.0177	0.0267	0.0345	0.0495	0.0652	0.0485	0.0415	0.0397
**2R**	0.0680	0.0278	0.0244	0.0258	0.0630	0.0462	0.0805	0.0586	0.0524	0.0493
**3L**	0.0451	0.0191	0.0221	0.0400	0.0291	0.0322	0.0563	0.0447	0.0361	0.0361
**3R**	0.0345	0.0210	0.0158	0.0327	0.0263	0.0380	0.0632	0.0431	0.0336	0.0343
**genome-wide**	0.0507	0.0239	0.0202	0.0307	0.0408	0.0421	0.0681	0.0495	0.0415	0.0407
	completed soft – linked *(proportion)* *corrected for false discovery*									
**2L**	0.1012	0.0822	0.1304	0.2419	0.1463	0.3396	0.3727	0.2036	0.1749	0.2022
**2R**	0.1180	0.1606	0.1281	0.2697	0.2234	0.2844	0.4559	0.1650	0.1942	0.2256
**3L**	0.0724	0.0839	0.1217	0.3541	0.0971	0.1781	0.2214	0.1454	0.1335	0.1592
**3R**	0.0716	0.0873	0.1278	0.3207	0.0721	0.2242	0.2529	0.1549	0.1414	0.1639
**genome-wide**	0.0934	0.1097	0.1273	0.2951	0.1425	0.2598	0.3385	0.1662	0.1544	0.1916
	partial hard *(proportion)* *corrected for false discovery*									
**2L**	0.0277	0.0103	0.0460	0.0330	0.1369	0.0331	0.0395	0.0425	0.0363	0.0461
**2R**	0.0359	0.0241	0.0551	0.0836	0.0909	0.0359	0.0276	0.0291	0.0359	0.0478
**3L**	0.0162	0.0071	0.0134	0.0113	0.1614	0.0074	0.0118	0.0114	0.0116	0.0300
**3R**	0.0231	0.0135	0.0384	0.0175	0.1667	0.0315	0.0193	0.0109	0.0212	0.0401
**genome-wide**	0.0272	0.0151	0.0409	0.0405	0.1341	0.0291	0.0248	0.0233	0.0282	0.0419
	partial hard – linked *(proportion)* *corrected for false discovery*									
**2L**	0.0748	0.0223	0.1378	0.0863	0.1325	0.0567	0.0512	0.0821	0.0785	0.0805
**2R**	0.0993	0.0767	0.1335	0.1535	0.1334	0.0866	0.0372	0.0530	0.0929	0.0966
**3L**	0.0611	0.0115	0.0457	0.0329	0.1867	0.0208	0.0248	0.0179	0.0289	0.0502
**3R**	0.0789	0.0353	0.1108	0.0652	0.1821	0.0727	0.0341	0.0200	0.0689	0.0749
**genome-wide**	0.0820	0.0419	0.1119	0.0914	0.1564	0.0651	0.0370	0.0429	0.0736	0.0786
	partial soft *(proportion)* *corrected for false discovery*									
**2L**	0.0816	0.0274	0.0533	0.1026	0.0468	0.0385	0.1127	0.0987	0.0674	0.0702
**2R**	0.0643	0.0388	0.0673	0.0530	0.0455	0.0280	0.0987	0.1114	0.0587	0.0634
**3L**	0.0576	0.0257	0.0645	0.0833	0.0484	0.0203	0.0742	0.1004	0.0610	0.0593
**3R**	0.0682	0.0285	0.0632	0.1540	0.0492	0.0251	0.0983	0.1182	0.0657	0.0756
**genome-wide**	0.0678	0.0312	0.0628	0.0979	0.0473	0.0279	0.0971	0.1088	0.0653	0.0676
	partial soft – linked *(proportion)* *corrected for false discovery*									
**2L**	0.1723	0.1157	0.2884	0.4666	0.1327	0.1322	0.3085	0.2866	0.2294	0.2379

**2R**	0.1501	0.2584	0.2687	0.4003	0.1806	0.1097	0.2546	0.3147	0.2565	0.2421
**3L**	0.1534	0.1259	0.2874	0.4603	0.1588	0.0874	0.1885	0.2433	0.1737	0.2132
**3R**	0.1571	0.1435	0.2951	0.3931	0.1887	0.1225	0.2225	0.2550	0.2056	0.2222
**genome-wide**	0.1572	0.1723	0.2836	0.4230	0.1696	0.1141	0.2446	0.2787	0.2085	0.2304
	neutral *(count)* *corrected for false discovery*									
**2L**	1,224.2	2,022.7	940.1	120.6	941.3	936.4	137.4	676.2	938.3	874.9
**2R**	1,919.5	1,866.2	1,508.9	53.4	1,115.0	1,828.0	187.0	1,231.0	1,369.9	1,213.6
**3L**	1,233.9	1,827.4	1,157.4	46.4	679.6	1,505.3	1,003.8	1,140.5	1,149.0	1,074.3
**3R**	2,021.8	2,633.2	1,401.3	71.9	1,146.7	1,848.1	1,189.6	1,609.5	1,505.4	1,490.3
**genome-wide**	6,399.3	8,349.5	5,007.8	292.3	3,882.6	6,117.8	2,517.8	4,657.1	4,832.5	4,653.0
	completed hard *(count)* *corrected for false discovery*									
**2L**	0.0	0.0	0.0	1.9	1.0	0.0	0.0	1.0	0.0	0.5
**2R**	4.0	2.0	1.0	-0.1	1.0	1.0	4.0	1.0	1.0	1.7
**3L**	0.0	1.0	0.0	0.0	0.0	0.0	2.0	0.0	0.0	0.4
**3R**	0.0	3.0	2.0	-0.1	2.0	1.0	1.0	3.0	1.5	1.5
**genome-wide**	4.0	6.0	3.0	1.7	4.0	2.0	7.0	5.0	4.0	4.1
	completed hard – linked *(count)* *corrected for false discovery*									
**2L**	1.0	-3.0	5.0	11.0	0.0	5.0	0.0	2.0	1.5	2.6
**2R**	19.0	4.3	6.0	17.0	7.0	4.0	8.0	11.0	7.5	9.5
**3L**	2.0	1.3	1.0	8.0	1.0	3.0	1.0	2.0	1.7	2.4
**3R**	10.0	4.7	8.0	3.0	1.0	0.0	0.0	5.0	3.9	4.0
**genome-wide**	32.0	7.3	20.0	39.0	9.0	12.0	9.0	20.0	16.0	18.5
	completed soft *(count)* *corrected for false discovery*									
**2L**	124.0	72.8	51.2	83.2	87.8	132.8	178.3	138.4	105.9	108.6
**2R**	284.4	125.9	114.5	128.2	268.5	207.0	353.0	271.7	237.7	219.1
**3L**	93.8	48.2	57.5	120.3	62.2	74.3	133.9	116.8	84.1	88.4
**3R**	123.6	82.6	63.8	146.9	96.1	144.4	242.9	175.0	134.0	134.4
**genome-wide**	625.8	329.6	287.0	478.6	514.5	558.6	908.0	701.9	536.6	550.5
	completed soft – linked *(count)* *corrected for false discovery*									
**2L**	251.7	231.8	377.5	753.3	372.2	912.2	1,019.5	580.9	479.2	562.4
**2R**	493.5	726.9	601.4	1,341.8	952.7	1,273.8	1,998.8	764.6	858.7	1,019.2
**3L**	150.6	211.2	316.6	1,064.0	207.4	410.8	526.9	380.2	348.4	408.4
**3R**	256.6	343.6	517.4	1,441.4	263.1	853.2	971.7	629.3	573.4	659.5
**genome-wide**	1,152.4	1,513.6	1,812.9	4,600.6	1,795.3	3,450.0	4,516.9	2,355.1	2,084.0	2,649.6
	partial hard *(count)* *corrected for false discovery*									
**2L**	68.8	29.0	133.1	102.8	348.2	89.0	108.0	121.3	105.4	125.0
**2R**	150.1	109.0	258.5	415.9	387.7	161.0	121.0	134.8	155.5	217.2
**3L**	33.8	18.0	34.8	33.9	345.0	17.0	28.0	29.9	31.8	67.5

**3R**	83.0	53.0	155.6	78.9	608.6	120.0	74.0	44.4	80.9	152.2
**genome-wide**	335.6	209.0	582.0	631.4	1,689.4	387.0	331.0	330.3	361.3	562.0
	partial hard – linked *(count)* *corrected for false discovery*									
**2L**	186.1	63.0	399.1	268.9	337.0	152.3	140.0	234.3	210.2	222.6
**2R**	415.3	347.0	626.5	763.9	569.0	387.7	163.0	245.8	401.5	439.8
**3L**	127.1	29.0	118.8	99.0	399.0	48.0	59.0	46.9	79.0	115.8
**3R**	282.9	139.0	448.6	292.9	665.0	276.6	131.0	81.4	279.8	289.7
**genome-wide**	1,011.4	578.0	1,593.0	1,424.7	1,970.0	864.5	493.0	608.3	938.0	1,067.9
	partial soft *(count)* *corrected for false discovery*									
**2L**	202.9	77.4	154.2	319.4	119.0	103.3	308.2	281.7	178.5	195.8
**2R**	269.1	175.7	315.8	264.0	194.0	125.2	432.9	516.5	266.5	286.7
**3L**	119.7	64.8	167.9	250.2	103.4	46.8	176.7	262.6	143.8	149.0
**3R**	244.7	112.3	256.0	692.3	179.5	95.5	377.7	480.2	250.3	304.8
**genome-wide**	836.4	430.2	893.8	1,525.9	596.0	370.8	1,295.5	1,541.0	865.1	936.2
	partial soft – linked *(count)* *corrected for false discovery*									
**2L**	428.4	326.3	834.8	1,452.9	337.6	355.1	843.7	818.1	623.2	674.6
**2R**	628.0	1,169.9	1,261.4	1,991.9	770.1	491.3	1,116.3	1,458.7	1,143.1	1,110.9
**3L**	319.2	317.0	747.9	1,383.3	339.4	201.7	448.7	636.3	394.1	549.2
**3R**	563.5	564.6	1,194.3	1,766.8	689.1	466.3	855.1	1,036.2	772.1	892.0
**genome-wide**	1,939.0	2,377.9	4,038.5	6,594.8	2,136.2	1,514.3	3,263.8	3,949.3	2,820.8	3,226.7

Table S4. Enrichments of selective sweeps within DNA regions for each empirical mosquito population dataset.

		AOM	BFM	BFS	CMS	GAS	GNS	GWA	UGS
		all four sweep states							
**gene**	**count**	1177	820	1173	1530	1636	1028	1698	1839
	**enrichment**	1.11	1.24	1.19	1.13	1.08	1.14	1.15	1.18
	***p*-value**	≤0.0001	≤0.0001	≤0.0001	≤0.0001	≤0.0001	≤0.0001	≤0.0001	≤0.0001
**mRNA**	**count**	1176	819	1171	1529	1634	1028	1697	1836
	**enrichment**	1.11	1.24	1.19	1.13	1.08	1.14	1.15	1.18
	***p*-value**	≤0.0001	≤0.0001	≤0.0001	≤0.0001	≤0.0001	≤0.0001	≤0.0001	≤0.0001
**exon**	**count**	919	700	951	1230	1280	824	1365	1506
	**enrichment**	1.18	1.44	1.31	1.23	1.14	1.24	1.25	1.31
	***p*-value**	≤0.0001	≤0.0001	≤0.0001	≤0.0001	≤0.0001	≤0.0001	≤0.0001	≤0.0001
**CDS**	**count**	887	685	930	1198	1231	798	1325	1468
	**enrichment**	1.19	1.48	1.36	1.26	1.16	1.26	1.28	1.35
	***p*-value**	≤0.0001	≤0.0001	≤0.0001	≤0.0001	≤0.0001	≤0.0001	≤0.0001	≤0.0001
**5' UTR**	**count**	338	265	358	445	446	302	507	537
	**enrichment**	1.24	1.56	1.41	1.28	1.16	1.30	1.34	1.34
	***p*-value**	≤0.0001	≤0.0001	≤0.0001	≤0.0001	≤0.0001	≤0.0001	≤0.0001	≤0.0001
**3' UTR**	**count**	312	247	343	414	416	278	440	502
	**enrichment**	1.34	1.70	1.59	1.38	1.26	1.40	1.37	1.47
	***p*-value**	≤0.0001	≤0.0001	≤0.0001	≤0.0001	≤0.0001	≤0.0001	≤0.0001	≤0.0001
		completed hard							
**gene**	**count**	1	5	2	2	2	1	4	4
	**enrichment**	0.45	1.68	1.30	1.71	0.93	0.95	1.15	1.51
	***p*-value**	0.9278	0.1034	0.5170	0.5043	0.7459	0.7780	0.5054	0.2250
**mRNA**	**count**	1	5	2	2	2	1	4	4
	**enrichment**	0.45	1.69	1.30	1.71	0.93	0.95	1.15	1.52
	***p*-value**	0.9276	0.1020	0.5143	0.5043	0.7436	0.7770	0.5038	0.2237
**exon**	**count**	1	3	2	2	2	1	4	4
	**enrichment**	0.59	1.35	1.78	2.45	1.28	1.28	1.50	2.09
	***p*-value**	0.8484	0.3910	0.3126	0.2786	0.5070	0.6298	0.2734	0.0762
**CDS**	**count**	1	3	2	2	2	1	4	4
	**enrichment**	0.62	1.42	1.87	2.61	1.35	1.34	1.58	2.21
	***p*-value**	0.8288	0.3574	0.2881	0.2549	0.4743	0.6087	0.2387	0.0647
**5' UTR**	**count**	0	2	1	0	1	0	3	1
	**enrichment**	0.00	2.56	2.55	0.00	1.80	0.00	3.17	1.48
	***p*-value**	1.0000	0.1804	0.3424	1.0000	0.4523	1.0000	0.0652	0.5172
**3' UTR**	**count**	0	2	1	0	1	1	0	2
	**enrichment**	0.00	2.94	2.92	0.00	2.13	4.15	0.00	3.50
	***p*-value**	1.0000	0.1430	0.3015	1.0000	0.3906	0.2259	1.0000	0.1017
		completed soft							
**gene**	**count**	485	345	283	376	425	521	715	617
	**enrichment**	1.25	1.70	1.83	1.57	1.41	1.36	1.41	1.47

	***p*-value**	<0.0001	<0.0001	<0.0001	<0.0001	<0.0001	<0.0001	<0.0001	<0.0001
**mRNA**	**count**	485	345	283	376	425	521	715	617
	**enrichment**	1.25	1.70	1.83	1.58	1.41	1.36	1.41	1.48
	***p*-value**	<0.0001	<0.0001	<0.0001	<0.0001	<0.0001	<0.0001	<0.0001	<0.0001
**exon**	**count**	424	335	276	356	394	459	665	582
	**enrichment**	1.47	2.24	2.39	1.99	1.76	1.62	1.77	1.88
	***p*-value**	<0.0001	<0.0001	<0.0001	<0.0001	<0.0001	<0.0001	<0.0001	<0.0001
**CDS**	**count**	416	334	274	351	389	453	653	575
	**enrichment**	1.52	2.35	2.51	2.08	1.83	1.68	1.83	1.96
	***p*-value**	<0.0001	<0.0001	<0.0001	<0.0001	<0.0001	<0.0001	<0.0001	<0.0001
**5' UTR**	**count**	157	126	96	124	141	172	239	213
	**enrichment**	1.55	2.40	2.37	2.01	1.78	1.74	1.83	1.96
	***p*-value**	<0.0001	<0.0001	<0.0001	<0.0001	<0.0001	<0.0001	<0.0001	<0.0001
**3' UTR**	**count**	153	131	98	131	137	163	245	208
	**enrichment**	1.77	2.93	2.86	2.46	2.03	1.93	2.20	2.26
	***p*-value**	<0.0001	<0.0001	<0.0001	<0.0001	<0.0001	<0.0001	<0.0001	<0.0001
		partial hard							
**gene**	**count**	180	99	358	375	845	210	189	206
	**enrichment**	0.99	0.89	1.13	1.09	0.98	1.01	1.05	1.12
	***p*-value**	0.5989	0.9251	0.0018	0.0364	0.7931	0.4632	0.2261	0.0305
**mRNA**	**count**	180	99	357	374	844	210	189	206
	**enrichment**	0.99	0.89	1.13	1.08	0.98	1.01	1.05	1.12
	***p*-value**	0.5896	0.9219	0.0020	0.0392	0.7849	0.4540	0.2215	0.0294
**exon**	**count**	116	74	250	290	612	141	127	153
	**enrichment**	0.86	0.90	1.08	1.13	0.96	0.92	0.96	1.13
	***p*-value**	0.9707	0.8584	0.1037	0.0141	0.8605	0.8407	0.6916	0.0487
**CDS**	**count**	110	69	244	282	584	130	122	150
	**enrichment**	0.86	0.88	1.11	1.16	0.97	0.90	0.98	1.18
	***p*-value**	0.9685	0.8856	0.0415	0.0060	0.8130	0.9043	0.6225	0.0169
**5' UTR**	**count**	39	30	108	103	210	50	48	53
	**enrichment**	0.82	1.01	1.30	1.09	0.97	0.92	1.02	1.10
	***p*-value**	0.9118	0.4890	0.0046	0.2032	0.6747	0.7255	0.4641	0.2741
**3' UTR**	**count**	42	26	101	105	189	42	34	50
	**enrichment**	1.04	1.03	1.44	1.33	1.01	0.91	0.86	1.25
	***p*-value**	0.4317	0.4620	0.0003	0.0053	0.4343	0.7325	0.8156	0.0725
		partial soft							
**gene**	**count**	511	371	530	777	364	296	790	1012
	**enrichment**	1.05	1.08	1.04	1.01	1.06	0.96	1.01	1.07
	***p*-value**	0.0684	0.0298	0.1025	0.3927	0.0761	0.8089	0.3293	0.0019
**mRNA**	**count**	510	370	529	777	363	296	789	1009
	**enrichment**	1.05	1.08	1.04	1.01	1.06	0.97	1.01	1.07
	***p*-value**	0.0692	0.0323	0.1045	0.3704	0.0809	0.7996	0.3228	0.0023
**exon**	**count**	378	288	423	582	272	223	569	767
	**enrichment**	1.06	1.13	1.12	1.02	1.07	0.98	0.99	1.10
	***p*-value**	0.0903	0.0066	0.0021	0.2958	0.0884	0.6337	0.6480	0.0014

**CDS**	**count**	360	279	410	563	256	214	546	739
	**enrichment**	1.06	1.16	1.15	1.05	1.06	0.99	1.00	1.12
	***p*-value**	0.0885	0.0023	0.0008	0.1591	0.1288	0.5577	0.5153	0.0004
**5' UTR**	**count**	142	107	153	218	94	80	217	270
	**enrichment**	1.14	1.21	1.17	1.11	1.07	1.02	1.09	1.11
	***p*-value**	0.0535	0.0244	0.0253	0.0802	0.2633	0.4512	0.0966	0.0348
**3' UTR**	**count**	117	88	143	178	89	72	161	242
	**enrichment**	1.10	1.17	1.28	1.05	1.18	1.07	0.94	1.17
	***p*-value**	0.1448	0.0834	0.0015	0.2652	0.0557	0.2863	0.7917	0.0051

Significant values (*i.e.* $p < 0.05$) underlined.

Table S5. Enrichments of selective sweeps within groupings of insecticide resistance genes for each empirical mosquito population dataset.

		AOM	BFM	BFS	CMS	GAS	GNS	GWA	UGS
		all four sweep states							
**metabolism**	**count**	67	58	60	88	69	57	75	95
	**enrichment**	1.57	2.16	1.50	1.65	1.25	1.61	1.35	1.57
	***p*-value**	0.0011	≤0.0001	0.0048	≤0.0001	0.0464	0.0018	0.0076	0.0001
**target site**	**count**	9	9	5	7	7	7	8	9
	**enrichment**	1.95	2.90	1.16	1.38	1.12	1.69	1.28	1.44
	***p*-value**	0.0132	0.0007	0.4434	0.2087	0.4439	0.0823	0.2509	0.1096
**behavior**	**count**	18	19	15	20	20	18	18	20
	**enrichment**	1.73	2.63	1.42	1.51	1.37	2.04	1.24	1.26
	***p*-value**	0.0507	0.0039	0.1619	0.1041	0.1518	0.0187	0.2480	0.2231
**cuticular**	**count**	2	1	2	6	5	2	5	7
	**enrichment**	0.82	0.60	0.87	1.77	1.49	1.02	1.62	1.94
	***p*-value**	0.7046	0.8049	0.6813	0.1215	0.2408	0.5782	0.1921	0.0603
		completed hard							
**metabolism**	**count**	0	0	2	0	0	0	0	1
	**enrichment**	0.00	0.00	26.63	0.00	0.00	0.00	0.00	8.64
	***p*-value**	1.0000	1.0000	0.0121	1.0000	1.0000	1.0000	1.0000	0.0932
**target site**	**count**	0	0	0	0	0	0	0	0
	**enrichment**	0.00	0.00	0.00	0.00	0.00	0.00	0.00	0.00
	***p*-value**	1.0000	1.0000	1.0000	1.0000	1.0000	1.0000	1.0000	1.0000
**behavior**	**count**	0	0	2	0	0	1	2	3
	**enrichment**	0.00	0.00	80.00	0.00	0.00	68.97	45.56	94.64
	***p*-value**	1.0000	1.0000	0.0047	1.0000	1.0000	0.0109	0.0076	0.0020
**cuticular**	**count**	0	0	0	0	0	0	0	0
	**enrichment**	0.00	0.00	0.00	0.00	0.00	0.00	0.00	0.00
	***p*-value**	1.0000	1.0000	1.0000	1.0000	1.0000	1.0000	1.0000	1.0000
		completed soft							
**metabolism**	**count**	32	23	18	23	23	32	45	33
	**enrichment**	1.93	2.68	2.62	2.12	1.78	1.97	2.13	1.87
	***p*-value**	0.0046	0.0008	0.0038	0.0036	0.0179	0.0021	≤0.0001	0.0053
**target site**	**count**	5	5	0	3	4	5	5	3
	**enrichment**	2.59	4.51	0.00	2.26	2.41	2.45	1.85	1.42
	***p*-value**	0.0298	0.0025	1.0000	0.1322	0.0701	0.0390	0.1085	0.3543
**behavior**	**count**	11	4	2	10	12	9	7	9
	**enrichment**	2.79	1.79	1.13	3.91	3.89	2.29	1.29	1.98
	***p*-value**	0.0180	0.2175	0.4666	0.0040	0.0025	0.0512	0.3263	0.0908
**cuticular**	**count**	1	0	0	0	1	2	5	4
	**enrichment**	1.06	0.00	0.00	0.00	1.54	2.24	4.42	3.84
	***p*-value**	0.5901	1.0000	1.0000	1.0000	0.4597	0.2233	0.0075	0.0307
		partial hard							

**metabolism**	**count**	11	8	18	36	32	10	8	24
	**enrichment**	1.40	1.77	1.39	2.58	1.02	1.27	1.16	3.27
	***p*-value**	0.1944	0.1293	0.1453	0.0002	0.4736	0.2850	0.3799	0.0001
**target site**	**count**	0	0	1	4	3	1	0	1
	**enrichment**	0.00	0.00	0.66	2.70	0.80	1.11	0.00	1.07
	***p*-value**	1.0000	1.0000	0.8033	0.0479	0.7734	0.6117	1.0000	0.6268
**behavior**	**count**	1	2	2	1	8	1	1	0
	**enrichment**	0.52	1.50	0.59	0.27	0.93	0.50	0.62	0.00
	***p*-value**	0.7638	0.3395	0.7553	0.9470	0.5637	0.7603	0.6956	1.0000
**cuticular**	**count**	0	1	0	1	5	0	0	1
	**enrichment**	0.00	4.10	0.00	1.43	2.37	0.00	0.00	1.95
	***p*-value**	1.0000	0.2027	1.0000	0.4852	0.0684	1.0000	1.0000	0.3747
		partial soft							
**metabolism**	**count**	25	27	25	31	19	16	27	45
	**enrichment**	1.22	1.86	1.14	1.04	1.30	1.22	0.86	1.15
	***p*-value**	0.2060	0.0095	0.2976	0.4337	0.1829	0.2658	0.7766	0.2125
**target site**	**count**	4	4	4	2	0	2	3	5
	**enrichment**	1.59	2.18	1.55	0.68	0.00	1.15	0.79	1.15
	***p*-value**	0.2227	0.0966	0.2479	0.8377	1.0000	0.5334	0.7865	0.4586
**behavior**	**count**	6	13	9	9	2	7	9	10
	**enrichment**	1.19	3.36	1.56	1.27	0.55	2.23	1.11	0.95
	***p*-value**	0.3837	0.0043	0.1779	0.3076	0.7908	0.0840	0.4277	0.5751
**cuticular**	**count**	1	0	2	5	0	0	0	3
	**enrichment**	0.82	0.00	1.58	2.36	0.00	0.00	0.00	1.32
	***p*-value**	0.6906	1.0000	0.3598	0.0728	1.0000	1.0000	1.0000	0.3991

Significant values (*i.e.* $p < 0.05$) underlined.
